# Environmental Chemical Diethylhexyl Phthalate Alters Intestinal Microbiota Community Structure and Metabolite Profile in Mice

**DOI:** 10.1128/mSystems.00724-19

**Published:** 2019-12-10

**Authors:** Ming Lei, Rani Menon, Sara Manteiga, Nicholas Alden, Carrie Hunt, Robert C. Alaniz, Kyongbum Lee, Arul Jayaraman

**Affiliations:** a Department of Chemical and Biological Engineering, Tufts University, Medford, Massachusetts, USA; b Artie McFerrin Department of Chemical Engineering, Texas A&M University, College Station, Texas, USA; c Department of Biomedical Engineering, Texas A&M University, College Station, Texas, USA; d Department of Microbial Pathogenesis and Immunology, College of Medicine, Texas Health Science Center, Texas A&M University, College Station, Texas, USA; University of California, San Diego

**Keywords:** autism, metabolomics, microbiota, phthalates

## Abstract

Exposure to environmental chemicals during windows of development is a potentially contributing factor in gut microbiota dysbiosis and linked to chronic diseases and developmental disorders. We used a community-level model of microbiota metabolism to investigate the effects of diethylhexyl phthalate (DEHP), a ubiquitous plasticizer implicated in neurodevelopmental disorders, on the composition and metabolite outputs of gut microbiota in young mice. Administration of DEHP by oral gavage increased the abundance of *Lachnoclostridium*, while decreasing *Clostridium sensu stricto*. Addition of DEHP to *in vitro*-cultured cecal microbiota increased the abundance of *Paenibacillus* and *Lachnoclostridium*. Untargeted metabolomics showed that DEHP broadly altered the metabolite profile in the culture. Notably, DEHP enhanced the production of *p*-cresol while inhibiting butyrate synthesis. Metabolic model-guided correlation analysis indicated that the likely sources of *p*-cresol are *Clostridium* species. Monoculture of Lachnoclostridium bolteae confirmed that it is capable of producing *p-*hydroxyphenylacetic acid, the immediate precursor of *p-*cresol, and that the species’ growth is enhanced upon DEHP exposure. Taken together, these findings suggest a model where DEHP increases production of *p*-cresol, a bacterial metabolite linked with neurodevelopmental disorders, by expanding the abundance of species that synthesize the metabolite’s precursor.

**IMPORTANCE** Several previous studies have pointed to environmental chemical exposure during windows of development as a contributing factor in neurodevelopmental disorders and correlated these disorders with microbiota dysbiosis; however, little is known about how the chemicals specifically alter the microbiota to interfere with development. The findings reported in this paper unambiguously establish that a pollutant linked with neurodevelopmental disorders can directly modify the microbiota to promote the production of a potentially toxic metabolite (*p*-cresol) that has also been correlated with neurodevelopmental disorders. Furthermore, we used a novel modeling strategy to identify the responsible enzymes and bacterial sources of this metabolite. To the best of our knowledge, the present study is the first to characterize the functional consequence of phthalate exposure on a developed microbiota. Our results suggest that specific bacterial pathways could be developed as diagnostic and therapeutic targets against health risks posed by ingestion of environmental chemicals.

## Abstract (precis)

Several previous studies have pointed to environmental chemical exposure during windows of development as a contributing factor in neurodevelopmental disorders and correlated these disorders with microbiota dysbiosis; however, little is known about how the chemicals specifically alter the microbiota to interfere with development. The findings reported in this paper unambiguously establish that a pollutant linked with neurodevelopmental disorders can directly modify the microbiota to promote the production of a potentially toxic metabolite (*p*-cresol) that has also been correlated with neurodevelopmental disorders. Furthermore, we used a novel modeling strategy to identify the responsible enzymes and bacterial sources of this metabolite. To the best of our knowledge, the present study is the first to characterize the functional consequence of phthalate exposure on a developed microbiota. Our results suggest that specific bacterial pathways could be developed as diagnostic and therapeutic targets against health risks posed by ingestion of environmental chemicals.

## INTRODUCTION

The mammalian gastrointestinal (GI) tract harbors microbial communities that impact a wide array of physiological functions, including digestion, immune system development, and defense against pathogens. Alterations in the microbiota composition leading to functional imbalance, or dysbiosis, have been linked to various chronic diseases and disorders, including inflammatory bowel disease (1), colorectal cancer (2), fatty liver disease (3), diabetes (4), and neurodevelopmental disorders (5). Factors known to cause dysbiosis include diet, infection, and use of antibiotics. In recent years, environmental chemicals have emerged as another factor contributing to alterations in the microbiota.

Exposure to biologically active synthetic chemicals present in household and industrial products, particularly during critical windows of development, has been shown to result in microbiota dysbiosis and correlate with various disorders of the immune and nervous systems (6). An environmental chemical that is pervasive in the environment due to its widespread use as a plasticizer is diethylhexyl phthalate (DEHP) (7). In vertebrate animals, DEHP impacts reproduction and development (8). A recent study found increased serum DEHP concentrations in children diagnosed with autism spectrum disorder (ASD) (9). Additionally, fecal samples from children diagnosed with ASD have elevated concentrations of bacterial metabolites such as *p*-cresol (10), pointing to a potential link between the health effects of DEHP exposure and the intestinal microbiota.

This link is supported by multiple studies with other environmental chemicals correlating microbiota dysbiosis with adverse effects of exposure. A study in mice showed that exposure to benzo[*a*]pyrene resulted in pronounced alterations of the intestinal microbiota, including a decrease in the abundance of Akkermansia muciniphila, and an increase in the levels of inflammatory indicators (11). Another recent study found that bisphenol A (BPA) exposure exacerbated the effects of chemically induced colitis and that these effects were accompanied by altered fecal levels of tryptophan-derived metabolites (12).

The importance of bacterially produced metabolites in dysbiosis-related disorders was highlighted by Hsiao et al., who showed that the behavioral abnormalities observed in a maternal immune activation (MIA) model of anxiety-like behavior in mice correlated with changes in the abundance of intestinal bacteria and the concentration of bacterial metabolites in serum (13). This study also showed that the behavioral abnormalities in the MIA model could be improved by controlling the level of a specific tyrosine metabolite, 4-ethylphenyl sulfate. Altered levels of microbiota-associated metabolites such as indoxyl sulfate and *p*-hydroxyphenyl lactate have also been detected in blood and urine of children diagnosed with ASD (14, 15), suggesting that the link between bacterial metabolites and neurodevelopmental disorders could be relevant in humans. On the other hand, it is unclear whether the aforementioned alterations in bacterial metabolite profiles directly result from environmental chemical exposure. While several studies have investigated the effects of DEHP exposure on host reproductive, nervous, and metabolic tissues (16–18), little is known about the impact of this ubiquitous chemical on intestinal microbiota composition and function.

A majority of studies have used early-life exposure models to study the effects of environmental chemicals on the intestinal microbiota. Observations from these studies suggest that changes to the intestinal microbiota can persist beyond the pre- or perinatal exposure period (19, 20). For example, perinatal exposure of rabbits to BPA *in utero* and during the first week of nursing led to a decrease in short-chain fatty acid (SCFA)-producing bacteria at 6 weeks of age (21). Another study found that continuous exposure to diethyl phthalate, methylparaben, and triclosan beginning at birth induced significant changes to the gut microbiota of adolescent rats (22). To date, few studies have looked at the effect of environmental chemical exposure on a developed microbiota.

While *in vivo* studies on the microbiota offer physiologically relevant insights, they can often be difficult to interpret due to confounding influences from the host (23). Apart from directly altering microbiota composition, environmental chemicals can also indirectly cause microbiota dysbiosis, for example, by bringing about intestinal inflammation through activation of host receptors (24). Moreover, receptor activation can occur through an intestinal or liver biotransformation product rather than the chemical itself (25). To elucidate the mechanistic role of dysbiosis resulting from environmental chemical exposure in a particular disease or disorder, it is important to delineate the effects of environmental chemical exposure on the intestinal microbiota from those on the host.

In this work, we used *in vivo* exposure in mice and an *in vitro* culture model to investigate the effect of DEHP on the intestinal microbiota composition and its metabolite output. Our results suggest that environmental chemical exposure can directly modify the intestinal microbiota to increase production of a potentially neurotoxic microbial metabolite linked with behavioral abnormalities.

## RESULTS

### Gut microbiota composition is altered *in vivo* in a time-dependent manner.

**FIG 1:**
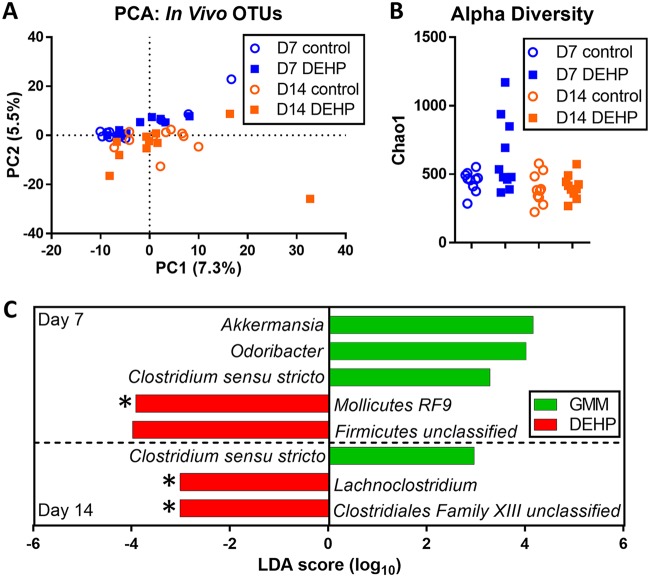
Metagenomic (16S rRNA) analysis of fecal microbiota from DEHP-exposed mice. (A) PCA on OTU counts. The percentages represent the percent variances explained by each axis. Alpha-diversity (B) and LefSe analysis (C) of fecal microbiota OTU counts. *, *P* < 0.05 by two-tailed *t* test.

We investigated the effect of DEHP exposure on the gut microbiota by administering the chemical to 6- to 8-week-old female C57BL/6 mice via oral gavage and analyzing the changes in the fecal microbial community at day 7 and day 14 postexposure using 16S rRNA sequencing. Principal-component analysis (PCA) of operational taxonomic unit (OTU) counts showed samples grouping together by time point but not by DEHP treatment (Fig. 1A), suggesting that changes in OTU profile driven by the host’s age may dominate over DEHP-driven changes. This trend agreed with classification results from partial least-squares discriminant analysis (PLS-DA), which achieved stronger separation between OTU profiles when samples were classified based on time point than chemical treatment (see Fig. S1 in the supplemental material). The results from PLS-DA also indicated that the effect of DEHP on the OTU profiles was greater on day 7 than day 14. Consistent with this observation, samples from DEHP-treated mice showed a higher alpha diversity (Chao1 index) than control mice on day 7 but not on day 14 (Fig. 1B).

10.1128/mSystems.00724-19.2 FIG S1 Scatter plot of latent variable (LV) scores from PLS-DA on OTU counts for fecal samples. *R*^2^X (cumulative) = 0.145, *R*^2^Y (cumulative) = 0.989, *Q*^2^ (cumulative) = 0.714, and root-mean-square error of estimation (RMSEE) = 0.124. Download FIG S1, EPS file, 0.4 MB. Copyright © 2019 Lei et al. 2019 Lei et al. This content is distributed under the terms of the Creative Commons Attribution 4.0 International license.

Comparisons of OTU counts at the genus level identified only a small number of significant differences at both time points (Fig. 1C). Linear discriminant analysis of the effect size (LefSe) indicated that *Akkermansia*, *Odoribacter*, and *Clostridium sensu stricto* decreased in the DEHP samples collected on day 7. Of these, only *Clostridium sensu stricto* was also decreased in the day-14 samples. An unclassified genus belonging to the order *Mollicutes* RF9 was increased in abundance in the DEHP samples on day 7, and *Lachnoclostridium* was increased on day 14. These differences were also significant by a two-tailed *t* test.

### Fecal metabolite profile is more strongly influenced by host-dependent factors than DEHP treatment.

**FIG 2:**
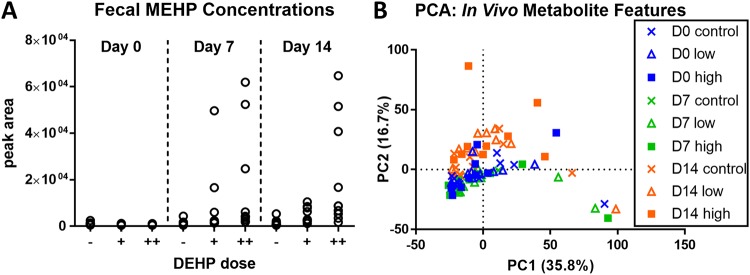
Metabolite analysis of fecal microbiota from DEHP-exposed mice. (A) LC-MS identification of MEHP in fecal material collected at days 7 and 14 from animals fed a low (+) or high (++) dose of DEHP. The level of MEHP in control samples (−) was below the limit of detection. (B) Scatter plot of the first two PC scores from PCA of the metabolite data.

To determine whether the phthalate exposure also altered the profile of intestinal metabolites, we analyzed the fecal material using untargeted liquid chromatography-mass spectrometry (LC-MS) metabolomics. We first confirmed that the orally administered DEHP was available to the microbiota by identifying the presence of mono(2-ethylhexyl)phthalate (MEHP), a product of enzyme-catalyzed DEHP degradation (26), in the metabolite data. As expected, MEHP was detected in fecal samples from DEHP-treated mice but not in control mice (Fig. 2A). Similar to the OTU profiles, results of PCA on the LC-MS features indicated that DEHP had a lesser effect on the fecal metabolite profile than the host’s age (Fig. 2B). This result was consistent with two-tailed *t* tests performed on individual data features, i.e., metabolites, which revealed very few statistically significant differences (none that could be assigned a putative identity) between time-matched samples from control and DEHP-treated mice on days 0, 7, and 14 (less than 1.6%, 2.3%, and 8.0% of total detected features, respectively). We detected a larger number of features that were significantly elevated or reduced in the day-14 fecal samples relative to that in day-0 and -7 samples (10.9% and 23.7%, respectively). These trends suggested that the global profile of fecal metabolites is more strongly influenced by host factors such as aging. To more directly assess the effects of DEHP on the microbiota in isolation from host influences, we performed the DEHP exposure experiment using an anaerobic batch culture model of murine cecal microbiota.

### Anaerobic cecal batch culture captures *in vivo* microbiota diversity.

**FIG 3:**
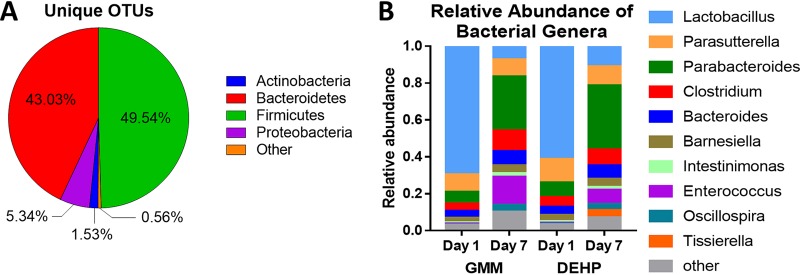
Metagenomic analysis of *in vitro* cecal luminal contents culture. (A) Phylum-level classification of unique OTUs in DEHP-treated cultures. (B) Relative abundance of bacterial genera on days 1 and 7 in control (GMM) and DEHP-treated cultures.

We assessed whether the cecal content culture could represent the biochemical diversity of murine cecal microbiota by characterizing the OTU profile of control cultures in gut microbiota medium (GMM) (27) without DEHP. Using QIIME with the SILVA database (28) as the reference, we identified approximately 2,000 distinct OTUs that are present on both days 1 and 7 of the culture. Nearly all of the OTUs (99.4%) belonged to four bacterial phyla: *Actinobacteria*, *Bacteroidetes*, *Firmicutes*, and *Proteobacteria* (Fig. 3A). The number of families and genera represented in the culture were 65 and 119, respectively. A comparison of OTUs detected on day 7 of cultured cecal contents with cecal contents harvested from 8-week-old female C57BL/6 mice showed 100% similarity in relative abundance at the phylum level and greater than 70% similarity at the genus and species levels (data not shown). From day 1 to 7, there were significant shifts in the relative abundances of the OTUs. At the genus level, *Lactobacillus* and *Parabacteroides* showed the maximum decrease and increase, respectively, in terms of OTU counts, whereas *Fluviicola* and *Enterococcus* showed the greatest decrease and increase, respectively, in terms of fold change (Fig. 3B).

### Cecal culture produces a diverse array of secondary and amino acid metabolites.

**FIG 4:**
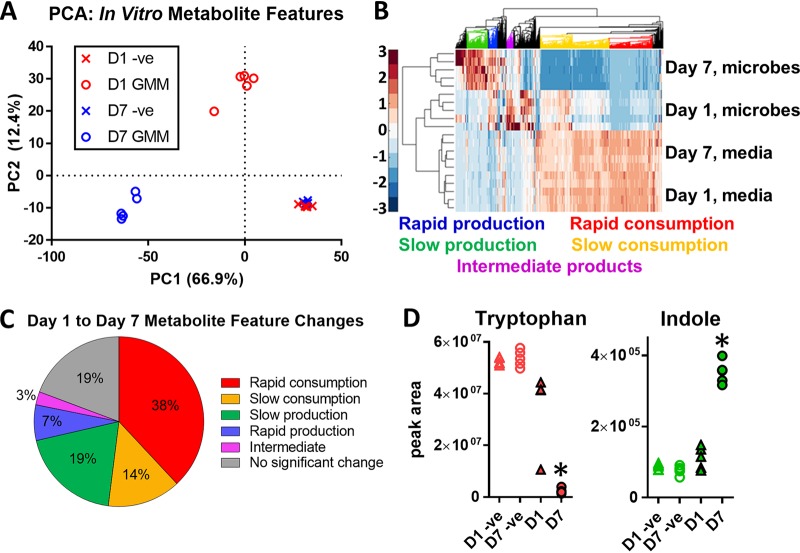
Metabolite profiles from *in vitro* cultured cecal luminal contents. (A) Scatter plot of the first two scores from PCA representing microbial metabolites produced on day 1 and day 7. (B) Heat map of detected ion peaks with different patterns of substrate utilization and product formation. (C) Percentage distribution of detected features classified as products, substrates, or intermediates based on their time profiles. (D) Profiles of tryptophan and indole in the cecal luminal content cultures. Filled and open markers represent inoculated cultures and tubes incubated without luminal contents, respectively. Triangles and circles represent day-1 and -7 time points, respectively. The colors correspond to the classifications in the heat map and pie chart. *, *P* < 0.05 compared to inoculated culture at day 1 (two-tailed *t* test).

We next characterized the major metabolic products and substrates of the cecal cultures using untargeted LC-MS experiments. Principal-component analysis (PCA) performed on the untargeted LC-MS features showed clear separation between day-1 and -7 samples from the inoculated GMM cultures (Fig. 4A). In contrast, day 1 and 7 samples from culture tubes containing GMM without cells grouped closely together. Hierarchical clustering of the LC-MS data features revealed five distinct patterns (Fig. 4B). The first group of features represented metabolites that were rapidly consumed and significantly depleted by day 1. A second larger group was consumed more slowly, with significant depletion occurring only by day 7. The third group comprised rapidly produced metabolites that were significantly elevated by day 1. The fourth group of metabolites was produced more slowly and was significantly elevated by day 7 but not day 1. The fifth group was significantly elevated by day 1 but reduced by day 7 (Fig. 4C).

Putative metabolite identities were assigned to the LC-MS data features based on accurate mass and product ion spectra (tandem mass spectrometry [MS/MS] data). Overall, 118 and 156 of the features in the positive- and negative-mode ionization data, respectively, were assigned a putative KEGG compound identifier. The merged list of annotated, i.e., putatively identified, metabolites from both ionization modes comprised 204 unique compounds (see Table S1). The overlap in metabolites identified by positive- and negative-mode experiments was very small (27/231), indicating that using two different LC-MS methods significantly broadened coverage.

10.1128/mSystems.00724-19.9 TABLE S1 Putatively identified compounds in cecal culture. Download Table S1, DOCX file, 0.04 MB. Copyright © 2019 Lei et al. 2019 Lei et al. This content is distributed under the terms of the Creative Commons Attribution 4.0 International license.

We utilized the Search Pathway tool of KEGG Mapper to associate each putatively identified metabolite with one or more functional categories. Based on this mapping, the largest KEGG function categories were biosynthesis of secondary metabolites (54 mapped metabolites), microbial metabolism in diverse environments (29), and biosynthesis of antibiotics (30). Additional pathways captured by the data included fermentative reactions known to occur in the intestine, such as l-carnitine metabolism to gamma-butyrobetaine and trimethylamine. When the different amino acid metabolism subcategories were pooled, more than one-third (79/204) of the metabolites belonged to this more general category. Interestingly, we detected the production of the neurotransmitter serotonin, despite the absence of any host cells in the cultures. Other aromatic amino acid (AAA) products, including the tryptophan metabolites indole (Fig. 4D), indole-3-propionic acid, and indole-3-pyruvic acid, were also detected. Phenylalanine-derived metabolites detected in the cultures included phenylacetic acid, phenylpropionic acid, and 3-(3-hydroxyphenyl)propionic acid and the tyrosine-derived metabolites detected included *p*-cresol and *p*-hydroxyphenylacetic acid.

### Metabolic function in cecal cultures is distributed heterogeneously across taxonomic groups.

**FIG 5:**
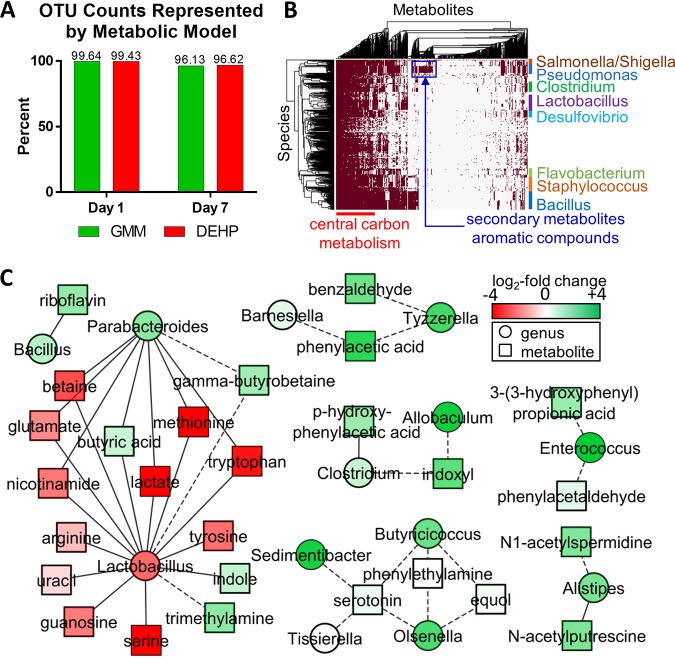
Model of metabolic reactions in *in vitro* culture of cecal luminal contents. (A) Fraction of genus-level OTU counts represented by the metabolic model. (B) Hierarchical clustering of genera and metabolites in the model. (C) Correlation network showing significant Pearson correlations (absolute PCC ≥ 0.76, *P* < 0.05) between genera (circles) and metabolites (squares). Fold change from days 1 to 7 is indicated by red (decrease) and green (increase) colors. Solid lines between nodes indicate that the genus has at least one species capable of metabolizing the connected metabolite (per database annotation of the genome), while dotted lines indicate a purely empirical correlation.

We next analyzed the genomes of bacterial groups detected in the culture to characterize the enzymatic reactions responsible for the metabolic products. Cross-referencing the list of identified OTUs against KEGG and UniProt, we obtained a model that included at least one annotated genome for 94 of 119 genera detected in the cultures, accounting for greater than 96% of the bacterial counts (Fig. 5A).

We evaluated the coverage of metabolic functions represented in the model by comparing the orthologs of the model against the orthologs predicted by Tax4Fun (31) using 16S sequence data, reference sequences in the SILVA database, and annotated genomes cataloged in KEGG as inputs. Based on KEGG orthology (KO) numbers, 83% of the gene functions predicted by Tax4Fun overlapped with the estimates from our model. When weighted by the relative abundance of the OTUs, we found a strong correlation between ortholog counts from Tax4Fun and our model (data not shown). Despite the similarity in functional coverage with our model, the Tax4Fun prediction included many additional organisms, most of which (more than 80%) did not match the OTUs classified by the SILVA analysis in the cecal culture. Thus, our model parsimoniously covered the biochemical diversity of the cecal culture without overpredicting the underlying taxonomic diversity.

The molecular functions represented by KO numbers were used to associate the bacterial species in our model with enzymatic reactions and their corresponding metabolites. The reactions distributed highly unevenly across the different genera (Fig. 5B). Of the more than 4,000 reactions, only 4 (involved in DNA replication) mapped to every genus. Approximately 9% (349/4034) of the reactions mapped to a single genus, indicating that certain metabolic functions (e.g., carotenoid biosynthesis, steroid hormone biosynthesis, methane metabolism, glycosphingolipid biosynthesis, and benzoate degradation) required the participation of particular genera. Similar to the trend for reactions in the model, the metabolites also distributed heterogeneously across the different organisms. Amino acids (e.g., phenylalanine and tryptophan), nucleosides/nucleotides (e.g., uridine and, uracil), and vitamins (e.g., riboflavin) associated with nearly ubiquitous reactions found in more than 90% of the genera, whereas certain fermentation products (e.g., benzaldehyde and indole-3-acetate) associated with rare reactions found in less than 50% of the genera.

### Correlation analysis finds significant associations between metabolite levels and relative abundance of organisms in the culture.

The annotations of compounds putatively identified from untargeted LC-MS analysis were confirmed (or rejected) by matching their retention time (RT) and/or MS/MS spectra to high-purity chemical standards, yielding a list of 57 confidently identified or confirmed metabolites (see Table S2). To identify significant associations between individual OTUs and metabolites, we calculated Pearson correlation coefficients (PCCs) between the peak area of each confidently identified or confirmed metabolite and the relative abundance of each highly abundant genus (>0.05% of total OTU counts) detected on both days 1 and 7. After correcting for false-discovery rate (FDR), we found 46 significant correlations. We mapped these correlations to the metabolic model described above to highlight different interactions between organisms and metabolites based on whether or not an organism possessed the enzyme to directly act on the correlated metabolite. The resulting correlation network is shown as connected nodes in a bipartite graph (Fig. 5C). Excluding common amino acids and GMM components, the strongest correlations were *Lactobacillus*-lactate (PCC = 0.98), *Butyricicoccus*-serotonin (PCC = 0.93) *Enterococcus*–3-(3-hydroxyphenyl) propionic acid (PCC = 0.92), *Clostridium*-indoxyl (PCC = 0.91), and *Alistipes*–*N*1-acetylspermidine (PCC = 0.88) (Fig. 5C).

10.1128/mSystems.00724-19.10 TABLE S2 Confidently identified (level 2) and confirmed metabolites (level 1). Confident identification (level 2) refers to metabolites matching a standard by at least two of the following measures: accurate mass, MS/MS (external reference standards), and retention time (RT). Confirmed identification (level 1) refers to metabolites matching a standard by at least two of the following measures: accurate mass, MS/MS (internal reference standards), and retention time (RT). Download Table S2, DOCX file, 0.02 MB. Copyright © 2019 Lei et al. 2019 Lei et al. This content is distributed under the terms of the Creative Commons Attribution 4.0 International license.

### DEHP induces changes in microbiota composition *in vitro*.

**FIG 6:**
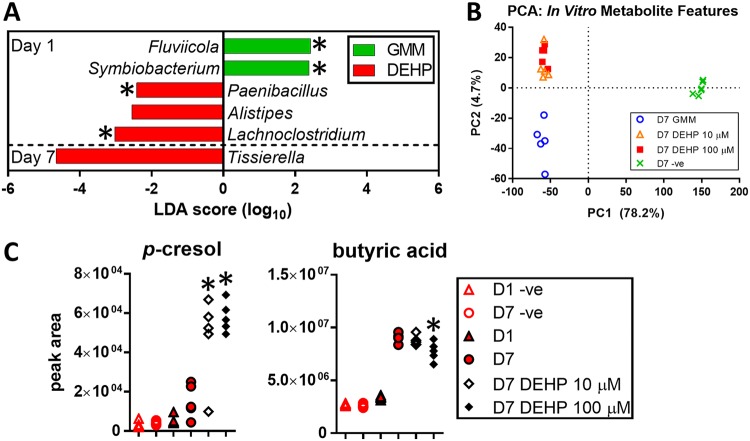
Significant microbial and metabolite changes in *in vitro*-cultured cecal luminal contents with DEHP. (A) LefSe analysis of genus-level microbiota changes induced by DEHP. *, *P* < 0.05 by two-tailed *t* test. (B) Scatter plot of first two PC scores from PCA of metabolite features detected in the cecal cultures (positive-mode IDA data). (C) Dose-dependent changes in *p*-cresol and butyric acid with DEHP on day 7. *, *P* < 0.05 compared to day 7 culture without DEHP addition (two-tailed *t* test).

We next examined the effects of DEHP on bacterial abundance in the cecal culture. At the genus level, DEHP increased the abundance of *Alistipes*, *Paenibacillus*, and *Lachnoclostridium* on day 1, while decreasing the abundance of *Fluviicola* and *Symbiobacterium*. On day 7, we detected an increase in *Tissierella* (Fig. 6A). At the OTU level, DEHP increased Alistipes putredinis, Lachnoclostridium bolteae, and Lachnoclostridium saccharolyticum on day 1. On day 7, we detected an increase in Tissierella praeacuta, and decreases in Bacillus velezensis and Lactobacillus brevis. Overall, DEHP exerted a greater effect on microbiota composition on day 1 than on day 7, in terms of both the number of altered OTUs and the changes in relative abundances of these OTUs.

### DEHP broadly alters metabolite profile of cecal microbiota.

Addition of DEHP significantly altered the profiles of metabolites in the cultures (Fig. 4A). At 10 and 100 μM, DEHP altered 16.8% and 20% of the LC-MS features, respectively, detected on day 7 in the positive ionization mode experiment. The negative-mode data showed even greater effects, with 46.7% and 47.2% of the detected features altered at 10 and 100 μM, respectively. Only a subset of these features were assigned chemical identities due to lack of matching entries in reference databases. Similarly to the *in vivo* exposure, we detected a dose-dependent accumulation of MEHP (see Fig. S2), confirming that the cecal culture is also capable of degrading DEHP under anaerobic conditions. Figure 4C shows representative profiles of confirmed metabolic products that were increased (cresol) and decreased (butyric acid) by DEHP treatment. *p*-Cresol was identified by matching both *m/z* and RT. We observed a shift in RT for samples compared to that for pure standards, possibly due to matrix effects of the samples. To account for this shift, we used local linear regression (see Fig. S3). To determine if the increase in *p*-cresol could be due to an organism that expanded in the culture upon DEHP exposure, we performed a monoculture of *L. bolteae* (see supplementary methods in Text S1), which increased in abundance upon DEHP exposure both *in vivo* and *in vitro*. The model-guided correlation analysis linked this organism with *p*-hydroxyphenylacetic acid, a direct precursor of *p*-cresol. Targeted LC-MS analysis of *L. bolteae* culture supernatant confirmed that the organism is capable of producing the *p*-cresol precursor. Moreover, *L. bolteae* in monoculture grew to greater optical density (OD) in medium supplemented with 100 μM DEHP than in control medium without DEHP (see Fig. S4), consistent with the findings from *in vivo* and cecal culture experiments.

10.1128/mSystems.00724-19.1 TEXT S1 Methods for Lachnoclostridium bolteae monoculture and untargeted LC-MS experiments. Download Text S1, DOCX file, 0.01 MB. Copyright © 2019 Lei et al. 2019 Lei et al. This content is distributed under the terms of the Creative Commons Attribution 4.0 International license.

10.1128/mSystems.00724-19.3 FIG S2 Conversion of DEHP to MEHP in cecal content culture. Error bars show one standard deviation (*N* = 5 independently inoculated cultures). +, 10 μM DEHP at the start of each batch cecal culture; ++, 100 μM DEHP at the start of each batch cecal culture; −, vehicle control; *, *P* < 0.05 compared to corresponding control samples by two-tailed *t* test. Download FIG S2, EPS file, 0.2 MB. Copyright © 2019 Lei et al. 2019 Lei et al. This content is distributed under the terms of the Creative Commons Attribution 4.0 International license.

10.1128/mSystems.00724-19.4 FIG S3 Local linear regression of sample retention time against retention time of pure standard. Download FIG S3, EPS file, 0.3 MB. Copyright © 2019 Lei et al. 2019 Lei et al. This content is distributed under the terms of the Creative Commons Attribution 4.0 International license.

10.1128/mSystems.00724-19.5 FIG S4 Production of *p*-hydroxyphenylacetic acid by *L. bolteae*. Bar graphs represent tyrosine concentration (A), *p*-hydroxyphenylacetic acid concentration (B), and cell density (C) after a 48-h incubation under anaerobic conditions. Data shown are means from 6 independently inoculated cultures, except vehicle controls. One vehicle control was incubated for each experimental group. Error bars represent one standard deviation. *, *P* < 0.05 compared to corresponding control samples by two-tailed *t* test. Download FIG S4, EPS file, 0.2 MB. Copyright © 2019 Lei et al. 2019 Lei et al. This content is distributed under the terms of the Creative Commons Attribution 4.0 International license.

An octadecenoic acid and an unknown octadecadienoic acid-specific isomer were decreased in the DEHP-treated cultures on day 7, while a metabolite putatively identified as isatin or indole-5,6-quinone was increased. An additional feature detected in positive-mode ionization at *m/z* 138.0886 had a dose-dependent increase in response to DEHP and was putatively identified as tyramine, 1-methylnicotinamide, or 2-hydroxyphenethylamine. It was not possible to assign a unique identity to this feature, because the product ion spectrum indicated that the corresponding ion chromatogram peak could represent more than one compound.

## DISCUSSION

Studies have implicated dysbiosis of the gut microbiota in developmental disorders associated with exposure to environmental toxicants (22, 32). In this work, we focus on the effects of DEHP, a pervasive environmental chemical and endocrine disruptor associated with neurodevelopmental disorders such as ASD (9). Previous *in vivo* studies (32, 33) used early-life exposure models to investigate the microbiota’s role in developmental health and disease. Fewer studies have investigated the effect of environmental chemical exposure on a developed microbial community in mammals. In the present study, we mimicked human exposure during adolescence by continuously exposing mice to DEHP from ages 6 to 8 weeks. Additionally, we used an anaerobic batch culture model to investigate the effects of DEHP on the microbiota community structure and metabolites.

Continuous DEHP exposure modestly increased the alpha-diversity of the fecal microbiota after 7 days, an effect that dissipated by day 14. While it is often assumed that a diverse gut microbiome is beneficial for the host, this is not necessarily the case, as gut health is also impacted by the microbial enterotype. After 14 days, mice exposed to DEHP showed an increased abundance of *Lachnoclostridium* and an unclassified genus of *Clostridiales* Family XIII. While the effect of DEHP on gut microbiota of young mice has not been previously reported, studies in humans have associated overrepresentation of *Lachnoclostridium* species with neurodevelopmental disorders such as ASD (34, 35). This suggests that the enterotype resulting from pollutant exposure could play a role in dysbiosis-associated neurodevelopmental disorders.

We detected minimal changes in metabolite profile of fecal material from mice exposed to DEHP for 7 or 14 days. This could be due to several factors. First, host-driven changes, e.g., due to development and age, could mask subtler effects of phthalate exposure. Previous studies have shown significant age-related changes in murine microbiome over a comparable (14-day) time period (36). In mice, the onset of puberty occurs at approximately 6 weeks of age. For young mice at 6 to 7 weeks of age, 2 weeks are equivalent to 3.8 to 5.4 human years of development (30). Second, microbial metabolites can be taken up and transformed by the host, which limits the extent to which fecal metabolite analysis can capture the profile of microbiota metabolites in the intestine. Transformation and elimination by the host also likely attenuate the impact of any potentially harmful metabolic products generated by the microbiota *in vivo*. Moreover, fecal metabolites comprise not only bacterial products but also dietary residues and endogenous metabolites produced by the host. To address these issues, we investigated the effect of DEHP exposure on gut microbiota community structure and biochemical function using an *in vitro* model.

*In vitro* models of the intestinal microbiota vary in their complexity, depending on the intestinal location being mimicked and the degree of biophysical detail being incorporated (37). In the present study, we used a relatively simple anaerobic batch culture model, as the focus was on capturing biochemical function. This model recapitulated up to 70% of the microbiota found in murine cecum at the genus level, including strict anaerobes. Importantly, the culture supported the production of metabolites typically associated with the fermentation of sugar and amino acid residues by intestinal bacteria. As the culture system lacks host cells, all of the accumulating products are unequivocally sourced from the detected bacteria. Among the detected products are potentially toxic compounds derived from AAAs, e.g., phenylethylamine (38) and phenylacetic acid (39). We also detected metabolites that are normally present at low levels in the intestines of healthy individuals but are elevated in developmental disorders. For example, 3-phenylpropionic acid and 3-(3-hydroxyphenyl)propionic acid are precursors of 3-(3-hydroxyphenyl)-3 hydroxypropionic acid (HPHPA), a compound elevated in the urine of children diagnosed with ASD (40). Another useful feature of the culture model is that the correlations identified between different metabolites as well as between metabolites and organisms can reveal the sources of particular metabolic products. For example, lactic acid accumulated in the culture by day 1 and was rapidly consumed by day 7, which correlated with a significant depletion in *Lactobacillus*, a known lactic acid producer. The depletion in lactic acid was significantly correlated with butyric acid production, consistent with previous reports on bacterial conversion of lactic acid to butyric acid *in vitro* (41). Taken together, these findings suggest that the anaerobic batch culture broadly captures representative metabolic functions of the murine gut microbiota while facilitating identification of bacterial metabolites and their source organisms. On the other hand, the *in vitro* model clearly cannot capture the impact of host contributions to the intestinal metabolite milieu, including transformation, absorption, and elimination.

We further analyzed the organism-metabolite correlations using a metabolic model to associate the correlations with enzymatic pathways encoded in the genomes of detected OTUs. Several of these associations confirm previously reported findings. For example, our analysis links *Bacillus* with riboflavin, consistent with previous reports on the synthesis of this vitamin by intestinal *Bacillus* species (42). Likewise, members of the genus *Alistipes* possess the enzyme putrescine *N*-acetyltransferase (EC 2.3.1.57), which converts putrescine into *N*-acetylputrescine (43). A third example is the production of *p*-cresol, which previous reports have attributed to *Clostridium* species (44). Our analysis links *Clostridium* to *p*-hydroxyphenylacetic acid, which is converted to *p*-cresol via *p*-hydroxyphenylacetate decarboxylase (45).

Not all of the significant correlations mapped to an enzyme in the metabolic model. One limitation of the model is that genome annotations in KEGG and UniProt are incomplete. For example, the annotations for *Parabacteroides* in the databases did not include an enzyme for butyric acid synthesis, but a recent study found *buk* (butyrate kinase) and *ptb* (phosphotransbutyrylase) in the genomes of intestinal *Parabacteroides* species (46). In this regard, correlations that do not map to a cataloged enzyme could facilitate the discovery of previously unknown metabolic functions of intestinal bacteria. Another example is a compound we putatively identified as either indoxyl or oxindole, which strongly correlated with the expansion of *Clostridium*. Currently known metabolic reactions that produce indoxyl or oxindole are catalyzed by monooxygenases requiring oxygen (47). As evidenced by the growth of obligate anaerobes, molecular oxygen is absent in the cultures, suggesting that there could be alternative mechanisms of incorporating hydroxyl groups into metabolites. Indeed, oxindole has been reported to be a product of anaerobic indole degradation (29, 48).

Gut microbes have an extensive capacity to break down xenobiotics, including environmental chemicals, which can modulate their toxicity and bioavailability in the host (49). As was the case *in vivo* (Fig. 2), we detected a dose-dependent accumulation of MEHP in the cecal content culture (see Fig. S2 in the supplemental material), indicating that organisms expressing the required esterase are also present *in vitro.* To confirm that MEHP was indeed the primary metabolic product, we performed additional targeted experiments to measure other potential degradation products of DEHP (50): 2-ethylhexanol, phthalic acid, mono(2-ethyl-5-hydroxyhexyl)phthalate (5OH-MEHP), mono(2-ethyl-5-oxohexyl)phthalate (5oxo-MEHP), mono(2-carboxymethylhexyl) phthalate (2cx-MMHP), 2-1(oxoethyl)-hexyl phthalate, 2-ethyl-4-oxy-hexyl phthalate, 2-carboxy-hexyl phthalate, 2-ethyl-3-carboxy-propylphthalate, and 2-ethyl-4-carboxy-butylphthalate. We did not detect any of these compounds in the cecal cultures (data not shown). While lack of detection does not prove the absence of these metabolites, the limit of detection of our assay places an upper bound on the concentrations of these other products at 10 nM.

Compared to that with *in vivo* exposure, we detected fewer changes in the microbiota composition upon DEHP addition to the culture medium. This is possibly due to the rapid degradation of DEHP, which was continuously administered to the mice but added as a bolus at the start of the culture. Nevertheless, we observed features common to both *in vivo* and *in vitro* exposures. Similar to that in the *in vivo* experiment, we detected an increase in *Lachnoclostridium*, although this increase was transient in the cultures. Additionally, we detected a transient increase in *Alistipes*, which has also been reported for subjects diagnosed with ASD and related GI conditions (51).

Treatment with DEHP significantly altered the profile of metabolic products accumulating in the culture. Notably, DEHP increased the accumulation of *p*-cresol, a putative biomarker of ASD (52), while decreasing the levels of butyric acid, a bacterial metabolite benefiting intestinal immune homeostasis and offering neuroprotective effects (53). The likely source of *p*-cresol is tyrosine metabolism by *Clostridium* species (54), although the specific strains responsible are not known. Correlation analysis on cecal cultures found a positive association between the genus *Clostridium* and *p*-hydroxyphenylacetic acid, the immediate precursor of *p*-cresol. We also determined that addition of DEHP to the culture medium increased the growth of *L. bolteae* (formerly classified as Clostridium bolteae) in both cecal culture and monoculture. Using targeted experiments, we confirmed that *L. bolteae* is indeed capable of producing *p*-hydroxyphenylacetic acid (Fig. S4). However, we did not detect *p*-cresol in monocultures of *L. bolteae*. This organism lacks hydroxyphenylacetate decarboxylase (Hpd), the enzyme that catalyzes the conversion of *p*-hydroxyphenylacetic to *p*-cresol. This suggests that other species are responsible for Hpd activity in the cecal culture. In a recent study, Saito et al. (55) screened more than 150 gut bacteria and found that approximately one-third of the strains can produce *p*-cresol. The highest producers are C. difficile, Romboutsia lituseburensis, Olsenella uli, and Blautia hydrogenotrophica; the latter three species all possess enzymes homologous to Hpd. In our study, we detected both *Olsenella* and *Blautia* species in the mixed culture, although neither genus showed significant expansion upon DEHP treatment. Taken together, these findings suggest a model where DEHP increases *p*-cresol production by expanding species that synthesize the immediate precursor (see Fig. S5). This also highlights the limitations of using monocultures (as we did with *L. bolteae*) to study the effect of an environmental chemical on the metabolic functions of the gut microbiota. Physiologically important biological responses may occur from cometabolism involving multiple species, even if the direct effect of a chemical targets one or a few species.

10.1128/mSystems.00724-19.6 FIG S5 Model of DEHP-induced increase in *p*-cresol production. Exposure to DEHP expands select species, e.g., *L. bolteae*, that synthesize *p*-hydroxyphenylacetate, the immediate precursor of *p*-cresol. The conversion of *p*-hydroxyphenylacetate to *p*-cresol likely takes place in other species, as *L. bolteae* lacks the required enzyme, hydroxyphenylacetate decarboxylase (Hpd). The enzyme has been detected in a number of gut bacteria, including species belonging to the genera *Olsenella* and *Blautia*, which were detected in the cecal culture. Download FIG S5, EPS file, 0.3 MB. Copyright © 2019 Lei et al. 2019 Lei et al. This content is distributed under the terms of the Creative Commons Attribution 4.0 International license.

In addition to the above-discussed metabolites, the untargeted analysis detected nearly 2,000 compounds whose amounts in the culture were significantly altered by DEHP in a dose-dependent fashion. This number likely overestimates the impact of DEHP on the intestinal metabolite profile, as the relatively simple batch culture model used in the present study lacks absorption, transformation, and elimination mechanisms active in the body. Only a small fraction (79/1,937) of these compounds could be assigned a putative identity, as many of the detected features’ MS/MS spectra could not be matched to available databases for annotation. This annotation is a necessary step toward subsequent confirmation using chemical standards. The total annotation rate (204/5408) achieved in the present study is comparable to those in previous metabolomics studies on the gut microbiota, which report annotation rates ranging from 2% (56) to 5% (57). The annotation method used in this study, which we described in a previous publication (58), takes into account multiple pieces of evidence when assigning a putative identity to a given LC-MS feature, including observations regarding metabolites that connect to the putatively identified metabolites by way of enzymatic reactions expected in the system under investigation. We have shown that this method substantially reduces the false-discovery rate compared to that with other commonly used annotation tools. On the other hand, this conservative approach left a large number of features as unannotated “dark matter.” Metabolite annotation and identification clearly remain bottlenecks in untargeted metabolomics, and further efforts are warranted to expand coverage of metabolites from commensal gut bacteria in spectral libraries.

The findings of the present study provide evidence that significant alterations could occur even in developed microbiota in response to environmental chemical exposure and that these alterations include overproduction of selected bacterial metabolites. Several of these metabolites have been found at elevated levels in urine or plasma of subjects diagnosed with neurodevelopmental disorders, in particular, ASD. Taken together with recent reports linking phthalate exposure and ASD, our findings suggest the intriguing possibility that the chemical could selectively modify the intestinal microbiota to promote the production of potentially toxic metabolites such as *p*-cresol. Whether metabolites such as *p*-cresol causally contribute to neurodevelopmental disorders or merely indicate dysbiosis associated with these disorders remains to be elucidated. Further work is warranted to determine whether earlier (e.g., immediately after birth) and longer term DEHP exposure would lead to more severe dysbiosis and affect behavioral outcomes.

## MATERIALS AND METHODS

### Materials.

All chemicals were purchased from Sigma-Aldrich (St. Louis, MO) unless otherwise specified. DEHP and MEHP were purchased from AccuStandard (New Haven, CT).

### DEHP exposure in mice.

Female C57BL/6J mice aged 4 to 5 weeks were purchased from Jackson Laboratories (Bar Harbor, ME) and maintained on an *ad libitum* chow diet (8604 Teklad Rodent diet; Envigo, Madison, WI). Mice were acclimatized to the animal facility for 1 week. At the start of the experiment, mice were randomly divided into two groups (DEHP and control; *n* = 10 each group). Mice belonging to the same treatment group were housed together (5 mice/cage). Mice were given either vehicle (corn oil) or a low or high dose of DEHP (1 or 10 mg/kg body weight/day) via oral gavage. Mice were gavaged with DEHP every other day. Fecal pellets were collected from each mouse immediately before the first gavage (day 0) and on days 7 and 14, flash frozen in liquid nitrogen, and stored at −80°C. On day 14, animals were euthanized via asphyxiation with CO_2_. Animals were handled in accordance with the Texas A&M University Health Sciences Center Institutional Animal Care and Use Committee guidelines under an approved animal use protocol (AUP IACUC 2017-0145).

### *In vitro* culture of cecal luminal contents.

Whole ceca from female C57BL/6J mice (6 to 8 weeks of age) were harvested and transported to an anaerobic chamber (Coy Lab, Grass Lake, MI) in an anaerobic transport medium (Anaerobe Systems, Morgan Hill, CA). Luminal contents were isolated from the ceca inside the chamber and then suspended in a slurry in 1 ml of prereduced phosphate-buffered saline (PBS) containing 0.1% cysteine by vortexing the suspension for 2 min. Gut microbiota medium (GMM) was prepared as described previously (27). Each batch of cecal luminal content slurry from a mouse was inoculated in a separate glass test tube containing 10 ml of GMM or GMM supplemented with a low or high dose of DEHP (10 or 100 μM). The inoculated tubes were incubated at 37°C for up to 7 days under anaerobic conditions. Tubes containing GMM but without inoculation and incubated under the same conditions were used as negative controls. Culture (or medium) samples were collected on days 1 and 7 postinoculation by removing test tubes from the incubator and centrifuging them at 13,000 × *g* for 10 min at 4°C. The cell pellet and supernatant were stored at −80°C for further analysis.

### Extraction of metabolites.

The fecal pellets and *in vitro* cecal luminal culture samples were homogenized using lysing matrix E beads (MO BIO, Carlsbad, CA) on a bead beater (VWR, Radnor, PA) with equal volumes of cold methanol and one-half volume of chloroform. The samples were homogenized for 1 min on the bead beater, cooled on ice for 1 min, and homogenized again for another 2 min. The samples were then centrifuged at 10,000 × *g* at 4°C for 10 min. The supernatant was filtered through a 70-μm sterile nylon cell strainer into a clean sample tube and mixed with 0.6 ml of ice-cold water using a vortex mixer. This mixture was centrifuged again at 10,000 × *g* for 5 min to obtain phase separation. The upper and lower phases were separately collected using a syringe while taking care not to disturb the interface. The upper phase was dried to a pellet using a Vacufuge (Eppendorf, Hauppauge, NY) and stored at −80°C until further analysis. Prior to LC-MS analysis, the dried samples were reconstituted in 50 μl of methanol/water (1:1 [vol/vol]).

### Untargeted metabolomics.

The extracted samples were analyzed for global metabolite profiles using information-dependent acquisition (IDA) experiments performed on a triple-quadrupole time-of-flight instrument (5600+; AB Sciex) coupled to a binary pump high-performance liquid chromatography (HPLC) system (1260 Infinity; Agilent). Each sample was analyzed twice, using two different combinations of LC methods and ionization modes to obtain broad coverage of metabolites having various polarities and isoelectric points (see supplementary methods in Text S1). Raw data were processed in MarkerView (v. 1.2; AB Sciex) to determine the ion peaks. The peaks were aligned based on *m/z* and retention time (RT) (10 ppm and 0.5 min tolerance, respectively) and then filtered based on intensity (100 cps threshold) to eliminate low-quality peaks. An additional filter was applied using MarkerView to retain only monoisotopic ions, i.e., represent a series of isotopologues by their corresponding monoisotopic *m/z*. The retained ions were organized into a feature table, with each feature specified by *m/z* and RT. In the case where a precursor ion detected by the time of flight (TOF) survey scan triggered an MS/MS scan, the corresponding MS/MS spectrum was extracted from the product ion scan data and added to the feature table. Each feature was searched against spectral libraries in METLIN (59), HMDB (60), and NIST (61). The MS/MS spectrum of each feature was also analyzed using *in silico* fragmentation tools MetFrag (62) and CFM-ID (63). These analyses identified several annotations for many of the features. To systematically determine the most likely identities for these features in the context of murine cecal microbiota metabolism, we applied an automated annotation procedure (BioCAn) that combines the outputs from the database searches and fragmentation analyses with a metabolic model (see below) for the biological system of interest (58). Briefly, BioCAn maps each unique mass in the feature table onto a metabolic network representing the enzymatic reactions possible in the system of interest and evaluates the likelihood a correct mapping between a detected mass and a metabolite in the network has occurred based on how many other metabolites in the neighborhood of the metabolite in question also map to a detected mass. The product ion spectra of annotated features were further inspected manually and matched against standards in METLIN, HMDB, and NIST to obtain a set of confidently identified metabolites. The annotations of these metabolites were confirmed (or rejected) by matching their RT and/or MS/MS spectra to those of pure standards run on the same instrument using the same method (see Fig. S6 and S7). Relative amounts of metabolites were quantified using MultiQuant 2.1 (AB Sciex) by manually integrating the corresponding peak areas in the extracted ion chromatograms (XICs).

10.1128/mSystems.00724-19.7 FIG S6 Mirror plots of sample (red) and standard (blue) MS/MS spectra for confidently identified and confirmed metabolites. *x* and *y* axes show the *m/z* values and relative intensity of each peak, respectively, in the MS/MS spectrum. Metabolites are listed in order of increasing *m/z*. Download FIG S6, EPS file, 0.3 MB. Copyright © 2019 Lei et al. 2019 Lei et al. This content is distributed under the terms of the Creative Commons Attribution 4.0 International license.

10.1128/mSystems.00724-19.8 FIG S7 Representative extracted ion chromatograms (±5 ppm) of metabolites with adjusted retention times based on local linear regression (see Fig. S3). Insets show MS/MS mirror plots. Metabolites are listed in order of increasing *m/z*. Download FIG S7, EPS file, 0.9 MB. Copyright © 2019 Lei et al. 2019 Lei et al. This content is distributed under the terms of the Creative Commons Attribution 4.0 International license.

### Targeted analysis of MEHP.

The fate of DEHP in the cecal culture was characterized by quantifying the amount of its major metabolic product, MEHP. Targeted analysis of MEHP utilized a product ion scan experiment as described previously (64).

### 16S rRNA sequencing analysis.

Fecal and *in vitro* cecal luminal culture pellets were homogenized, and microbial DNA was extracted from the homogenate using the standard protocol for the Power soil DNA extraction kit (MO BIO). The V4 region of 16S rRNA was sequenced on a MiSeq Illumina platform using protocols for paired-end sequencing from Kozhich et al. (65) at the Microbial Analysis, Resources, and Services (MARS) core facility at the University of Connecticut. Sequence reads were quality filtered, denoised, joined, chimera filtered, aligned, and classified using QIIME (66, 67). The SILVA database (28) was used for alignment and classification (97% similarity) of the OTUs. The OTU counts were normalized by subsampling to the lowest number of OTUs found in the sample.

### Metabolic model.

The OTU tables from QIIME analysis were used to build a metabolic model linking bacterial groups detected in the cecal cultures to metabolites that can be produced by these groups. To select species for inclusion in the model, we tabulated the most abundant OTUs detected in all samples from both days 1 and 7 of GMM culture, with a 0.01% cutoff for relative abundance. The genera associated with these OTUs were searched against the KEGG Organisms database to compile a list of organisms that have a complete genome sequence and an assigned KEGG organism code (68). This list was then manually curated to remove species unlikely to be present in murine cecum (e.g., soil-dwelling bacteria and extremophiles) by searching a microbiome database (69) and carefully examining the published literature. From this curated list, we generated a matrix linking an organism to reactions encoded by its genome. First, the KEGG Orthology identifiers (K numbers) and Enzyme Commission (EC) numbers associated with the organism codes were collected using the KEGG REST API. These K and EC numbers were then linked to KEGG reaction identifiers (R numbers). The linkages between organism codes and R numbers were arranged into an organism-reaction (OR_KEGG_) matrix, where each element (*i*, *j*) denotes the presence (“1”) or absence (“0”) of a reaction *i* in organism *j*.

The organisms in OR_KEGG_ accounted for 48 of the 119 most abundant genera in the cecal cultures. The remaining 71 genera were searched against the UniProt database to determine if high-quality genome sequences with functional annotations were available for any of the member strains. After removing species that are unlikely to be present in the murine cecum, organisms with high-quality functional annotations were added to an organism-enzyme matrix (OE_UniProt_), where each element (*i*, *j*) denotes the presence (“1”) or absence (“0”) of an enzyme *i* in organism *j*. The amino acid sequences from each of the remaining organisms lacking annotated genomes were downloaded from GenBank and assigned K numbers using BlastKOALA (70). The resulting linkages between organisms and K numbers were arranged into an organism-orthology matrix (OK_UniProt_). The K and EC numbers of these two matrices were linked to R numbers to generate a second organism-reaction matrix (OR_UniProt_). The two matrices OR_KEGG_ and OR_UniProt_ were combined to produce a final organism-reaction matrix (OR) for all detected genera with member species that have high-quality genome sequences. The metabolites associated with each organism were found by linking the reactions with their primary substrate-product pairs as defined by KEGG’s RCLASS data.

### Statistical analysis.

OTUs observed only once across all samples were filtered prior to PCA and PLS-DA in MATLAB (v. R2018a). Linear discriminant analysis of the effect size (LefSe) was used to characterize differences in the OTU counts between samples (71). Effects were considered statistically significant if they were assigned a *q* value of less than 0.05. A two-tailed *t* test with a cutoff *P* value of 0.05 was used to test for statistical significance of differences in OTU counts and metabolite levels between treatment groups. Pearson correlation coefficients (PCCs) were calculated between OTU counts (relative abundance) and peak areas of metabolites. Statistical significance of the PCCs was determined based on *P* values calculated using a two-tailed *t* test and corrected for false-discovery rate using the Benjamini-Hochberg (B-H) method (72). Statistically significant correlations (B-H adjusted *P* value < 0.05) between OTUs (at the level of genus) and metabolites were visualized in Cytoscape (v. 3.0).

## References

[1] Tamboli C, Neut C, Desreumaux P, Colombel J 2004 Dysbiosis in inflammatory bowel disease. Gut 53:1–4. doi:10.1136/gut.53.1.1. 14684564 PMC1773911

[2] Sobhani I, Tap J, Roudot-Thoraval F, Roperch JP, Letulle S, Langella P, Corthier G, Van Nhieu JT, Furet JP 2011 Microbial dysbiosis in colorectal cancer (CRC) patients. PLoS One 6:e16393. doi:10.1371/journal.pone.0016393. 21297998 PMC3029306

[3] Hoyles L, Fernandez-Real JM, Federici M, Serino M, Abbott J, Charpentier J, Heymes C, Luque JL, Anthony E, Barton RH, Chilloux J, Myridakis A, Martinez-Gili L, Moreno-Navarrete JM, Benhamed F, Azalbert V, Blasco-Baque V, Puig J, Xifra G, Ricart W, Tomlinson C, Woodbridge M, Cardellini M, Davato F, Cardolini I, Porzio O, Gentileschi P, Lopez F, Foufelle F, Butcher SA, Holmes E, Nicholson JK, Postic C, Burcelin R, Dumas ME 2018 Molecular phenomics and metagenomics of hepatic steatosis in non-diabetic obese women. Nat Med 24:1070–1080. doi:10.1038/s41591-018-0061-3. 29942096 PMC6140997

[4] Qin J, Li Y, Cai Z, Li S, Zhu J, Zhang F, Liang S, Zhang W, Guan Y, Shen D, Peng Y, Zhang D, Jie Z, Wu W, Qin Y, Xue W, Li J, Han L, Lu D, Wu P, Dai Y, Sun X, Li Z, Tang A, Zhong S, Li X, Chen W, Xu R, Wang M, Feng Q, Gong M, Yu J, Zhang Y, Zhang M, Hansen T, Sanchez G, Raes J, Falony G, Okuda S, Almeida M, LeChatelier E, Renault P, Pons N, Batto J-M, Zhang Z, Chen H, Yang R, Zheng W, Li S, Yang H, 2012 A metagenome-wide association study of gut microbiota in type 2 diabetes. Nature 490:55. doi:10.1038/nature11450. 23023125

[5] Rogers G, Keating D, Young R, Wong M, Licinio J, Wesselingh S 2016 From gut dysbiosis to altered brain function and mental illness: mechanisms and pathways. Mol Psychiatry 21:738. doi:10.1038/mp.2016.50. 27090305 PMC4879184

[6] Rosenfeld CS 2017 Gut dysbiosis in animals due to environmental chemical exposures. Front Cell Infect Microbiol 7:396–396. doi:10.3389/fcimb.2017.00396. 28936425 PMC5596107

[7] Erythropel HC, Maric M, Nicell JA, Leask RL, Yargeau V 2014 Leaching of the plasticizer di(2-ethylhexyl)phthalate (DEHP) from plastic containers and the question of human exposure. Appl Microbiol Biotechnol 98:9967–9981. doi:10.1007/s00253-014-6183-8. 25376446

[8] Kay VR, Chambers C, Foster WG 2013 Reproductive and developmental effects of phthalate diesters in females. Crit Rev Toxicol 43:200–219. doi:10.3109/10408444.2013.766149. 23405971 PMC3604737

[9] Kardas F, Bayram AK, Demirci E, Akin L, Ozmen S, Kendirci M, Canpolat M, Oztop DB, Narin F, Gumus H, Kumandas S, Per H 2016 Increased serum phthalates (MEHP, DEHP) and bisphenol A concentrations in children with autism spectrum disorder: the role of endocrine disruptors in autism etiopathogenesis. J Child Neurol 31:629–635. doi:10.1177/0883073815609150. 26450281

[10] Kang DW, Ilhan ZE, Isern NG, Hoyt DW, Howsmon DP, Shaffer M, Lozupone CA, Hahn J, Adams JB, Krajmalnik-Brown R 2018 Differences in fecal microbial metabolites and microbiota of children with autism spectrum disorders. Anaerobe 49:121–131. doi:10.1016/j.anaerobe.2017.12.007. 29274915

[11] Ribiere C, Peyret P, Parisot N, Darcha C, Dechelotte PJ, Barnich N, Peyretaillade E, Boucher D 2016 Oral exposure to environmental pollutant benzo[*a*]pyrene impacts the intestinal epithelium and induces gut microbial shifts in murine model. Sci Rep 6:31027. doi:10.1038/srep31027. 27503127 PMC4977522

[12] DeLuca JA, Allred KF, Menon R, Riordan R, Weeks BR, Jayaraman A, Allred CD 2018 Bisphenol-A alters microbiota metabolites derived from aromatic amino acids and worsens disease activity during colitis. Exp Biol Med (Maywood) 243:864–875. doi:10.1177/1535370218782139. 29874946 PMC6022909

[13] Hsiao EY, McBride SW, Hsien S, Sharon G, Hyde ER, McCue T, Codelli JA, Chow J, Reisman SE, Petrosino JF, Patterson PH, Mazmanian SK 2013 Microbiota modulate behavioral and physiological abnormalities associated with neurodevelopmental disorders. Cell 155:1451–1463. doi:10.1016/j.cell.2013.11.024. 24315484 PMC3897394

[14] West PR, Amaral DG, Bais P, Smith AM, Egnash LA, Ross ME, Palmer JA, Fontaine BR, Conard KR, Corbett BA, Cezar GG, Donley EL, Burrier RE 2014 Metabolomics as a tool for discovery of biomarkers of autism spectrum disorder in the blood plasma of children. PLoS One 9:e112445. doi:10.1371/journal.pone.0112445. 25380056 PMC4224480

[15] Dieme B, Mavel S, Blasco H, Tripi G, Bonnet-Brilhault F, Malvy J, Bocca C, Andres CR, Nadal-Desbarats L, Emond P 2015 Metabolomics study of urine in autism spectrum disorders using a multiplatform analytical methodology. J Proteome Res 14:5273–5282. doi:10.1021/acs.jproteome.5b00699. 26538324

[16] Hannon PR, Niermann S, Flaws JA 2016 Acute exposure to di(2-ethylhexyl) phthalate in adulthood causes adverse reproductive outcomes later in life and accelerates reproductive aging in female mice. Toxicol Sci 150:97–108. doi:10.1093/toxsci/kfv317. 26678702 PMC5009616

[17] Klöting N, Hesselbarth N, Gericke M, Kunath A, Biemann R, Chakaroun R, Kosacka J, Kovacs P, Kern M, Stumvoll M, Fischer B, Rolle-Kampczyk U, Feltens R, Otto W, Wissenbach DK, von Bergen M, Blüher M 2015 Di-(2-ethylhexyl)-phthalate (DEHP) causes impaired adipocyte function and alters serum metabolites. PLoS One 10:e0143190. doi:10.1371/journal.pone.0143190. 26630026 PMC4668085

[18] Holahan MR, Smith CA, Luu BE, Storey KB 2018 Preadolescent phthalate (DEHP) exposure is associated with elevated locomotor activity and reward-related behavior and a reduced number of tyrosine hydroxylase positive neurons in post-adolescent male and female rats. Toxicol Sci 165:512–530. doi:10.1093/toxsci/kfy171. 29982774

[19] Leclercq S, Mian FM, Stanisz AM, Bindels LB, Cambier E, Ben-Amram H, Koren O, Forsythe P, Bienenstock J 2017 Low-dose penicillin in early life induces long-term changes in murine gut microbiota, brain cytokines and behavior. Nat Commun 8:15062. doi:10.1038/ncomms15062. 28375200 PMC5382287

[20] Joly Condette C, Bach V, Mayeur C, Gay-Quéheillard J, Khorsi-Cauet H 2015 Chlorpyrifos exposure during perinatal period affects intestinal microbiota associated with delay of maturation of digestive tract in rats. J Pediatr Gastroenterol Nutr 61:30–40. doi:10.1097/MPG.0000000000000734. 25643018

[21] Reddivari L, Veeramachaneni DNR, Walters WA, Lozupone C, Palmer J, Hewage MKK, Bhatnagar R, Amir A, Kennett MJ, Knight R, Vanamala J 2017 Perinatal bisphenol A exposure induces chronic inflammation in rabbit offspring via modulation of gut bacteria and their metabolites. mSystems 2:e00093-17. doi:10.1128/mSystems.00093-17. 29034330 PMC5634791

[22] Hu J, Raikhel V, Gopalakrishnan K, Fernandez-Hernandez H, Lambertini L, Manservisi F, Falcioni L, Bua L, Belpoggi F, L Teitelbaum S, Chen J 2016 Effect of postnatal low-dose exposure to environmental chemicals on the gut microbiome in a rodent model. Microbiome 4:26. doi:10.1186/s40168-016-0173-2. 27301250 PMC4906585

[23] Spor A, Koren O, Ley R 2011 Unravelling the effects of the environment and host genotype on the gut microbiome. Nat Rev Microbiol 9:279–290. doi:10.1038/nrmicro2540. 21407244

[24] Zhang L, Nichols RG, Correll J, Murray IA, Tanaka N, Smith PB, Hubbard TD, Sebastian A, Albert I, Hatzakis E, Gonzalez FJ, Perdew GH, Patterson AD 2015 Persistent organic pollutants modify gut microbiota-host metabolic homeostasis in mice through aryl hydrocarbon receptor activation. Environ Health Perspect 123:679–688. doi:10.1289/ehp.1409055. 25768209 PMC4492271

[25] Zhang LS, Davies SS 2016 Microbial metabolism of dietary components to bioactive metabolites: opportunities for new therapeutic interventions. Genome Med 8:46. doi:10.1186/s13073-016-0296-x. 27102537 PMC4840492

[26] Tomita I, Nakamura Y, Yagi Y, Tutikawa K 1986 Fetotoxic effects of mono-2-ethylhexyl phthalate (MEHP) in mice. Environ Health Perspect 65:249–254. doi:10.1289/ehp.8665249. 3709449 PMC1474685

[27] Goodman AL, Kallstrom G, Faith JJ, Reyes A, Moore A, Dantas G, Gordon JI 2011 Extensive personal human gut microbiota culture collections characterized and manipulated in gnotobiotic mice. Proc Natl Acad Sci U S A 108:6252–6257. doi:10.1073/pnas.1102938108. 21436049 PMC3076821

[28] Quast C, Pruesse E, Yilmaz P, Gerken J, Schweer T, Yarza P, Peplies J, Glockner FO 2013 The SILVA ribosomal RNA gene database project: improved data processing and web-based tools. Nucleic Acids Res 41:D590–D596. doi:10.1093/nar/gks1219. 23193283 PMC3531112

[29] Ma Q, Zhang X, Qu Y 2018 Biodegradation and biotransformation of indole: advances and perspectives. Front Microbiol 9:2625. doi:10.3389/fmicb.2018.02625. 30443243 PMC6221969

[30] Dutta S, Sengupta P 2016 Men and mice: relating their ages. Life Sci 152:244–248. doi:10.1016/j.lfs.2015.10.025. 26596563

[31] Aßhauer KP, Wemheuer B, Daniel R, Meinicke P 2015 Tax4Fun: predicting functional profiles from metagenomic 16S rRNA data. Bioinformatics 31:2882–2884. doi:10.1093/bioinformatics/btv287. 25957349 PMC4547618

[32] Javurek AB, Spollen WG, Johnson SA, Bivens NJ, Bromert KH, Givan SA, Rosenfeld CS 2016 Effects of exposure to bisphenol A and ethinyl estradiol on the gut microbiota of parents and their offspring in a rodent model. Gut Microbes 7:471–485. doi:10.1080/19490976.2016.1234657. 27624382 PMC5103659

[33] Wu J, Wen XW, Faulk C, Boehnke K, Zhang H, Dolinoy DC, Xi C 2016 Perinatal lead exposure alters gut microbiota composition and results in sex-specific bodyweight increases in adult mice. Toxicol Sci 151:324–333. doi:10.1093/toxsci/kfw046. 26962054 PMC4880136

[34] Song Y, Liu C, Finegold SM 2004 Real-time PCR quantitation of clostridia in feces of autistic children. Appl Environ Microbiol 70:6459–6465. doi:10.1128/AEM.70.11.6459-6465.2004. 15528506 PMC525120

[35] Luna RA, Oezguen N, Balderas M, Venkatachalam A, Runge JK, Versalovic J, Veenstra-VanderWeele J, Anderson GM, Savidge T, Williams KC 2017 Distinct microbiome-neuroimmune signatures correlate with functional abdominal pain in children with autism spectrum disorder. Cell Mol Gastroenterol Hepatol 3:218–230. doi:10.1016/j.jcmgh.2016.11.008. 28275689 PMC5331780

[36] Zhao Y, Wu J, Li JV, Zhou NY, Tang H, Wang Y 2013 Gut microbiota composition modifies fecal metabolic profiles in mice. J Proteome Res 12:2987–2999. doi:10.1021/pr400263n. 23631562

[37] Van de Wiele T, Van den Abbeele P, Ossieur W, Possemiers S, Marzorati M 2015 The simulator of the human intestinal microbial ecosystem (SHIME), p 305–317. In Verhoeckx K, Cotter P, Lopez-Exposito I, Kleiveland C, Lea T, Mackie A, Requena T, Swiatecka D, Wichers H (ed), The impact of food bioactives on health: in vitro and ex vivo models. Springer, Cham, Switzerland. doi:10.1007/978-3-319-16104-4_27. 29787039

[38] Marcobal A, De las Rivas B, Landete JM, Tabera L, Muñoz R 2012 Tyramine and phenylethylamine biosynthesis by food bacteria. Crit Rev Food Sci Nutr 52:448–467. doi:10.1080/10408398.2010.500545. 22369263

[39] Ramezani A, Massy ZA, Meijers B, Evenepoel P, Vanholder R, Raj DS 2016 Role of the gut microbiome in uremia: a potential therapeutic target. Am J Kidney Dis 67:483–498. doi:10.1053/j.ajkd.2015.09.027. 26590448 PMC5408507

[40] Shaw W 2010 Increased urinary excretion of a 3–(3-hydroxyphenyl)-3-hydroxypropionic acid (HPHPA), an abnormal phenylalanine metabolite of *Clostridia* spp. in the gastrointestinal tract, in urine samples from patients with autism and schizophrenia. Nutr Neurosci 13:135–143. doi:10.1179/147683010X12611460763968. 20423563

[41] Duncan SH, Louis P, Flint HJ 2004 Lactate-utilizing bacteria, isolated from human feces, that produce butyrate as a major fermentation product. Appl Environ Microbiol 70:5810–5817. doi:10.1128/AEM.70.10.5810-5817.2004. 15466518 PMC522113

[42] LeBlanc JG, Milani C, de Giori GS, Sesma F, van Sinderen D, Ventura M 2013 Bacteria as vitamin suppliers to their host: a gut microbiota perspective. Curr Opin Biotechnol 24:160–168. doi:10.1016/j.copbio.2012.08.005. 22940212

[43] Liu R, Li Q, Ma R, Lin X, Xu H, Bi K 2013 Determination of polyamine metabolome in plasma and urine by ultrahigh performance liquid chromatography-tandem mass spectrometry method: application to identify potential markers for human hepatic cancer. Anal Chim Acta 791:36–45. doi:10.1016/j.aca.2013.06.044. 23890604

[44] Dawson LF, Donahue EH, Cartman ST, Barton RH, Bundy J, McNerney R, Minton NP, Wren BW 2011 The analysis of *para*-cresol production and tolerance in *Clostridium difficile* 027 and 012 strains. BMC Microbiol 11:86. doi:10.1186/1471-2180-11-86. 21527013 PMC3102038

[45] Moss CW, Hatheway CL, Lambert MA, McCroskey LM 1980 Production of phenylacetic and hydroxyphenylacetic acids by *Clostridium botulinum* type G. J Clin Microbiol 11:743–745. 7000821 10.1128/jcm.11.6.743-745.1980 PMC273498

[46] Hwang N, Eom T, Gupta SK, Jeong SY, Jeong DY, Kim YS, Lee JH, Sadowsky MJ, Unno T 2017 Genes and gut bacteria involved in luminal butyrate reduction caused by diet and loperamide. Genes (Basel) 8:E350. doi:10.3390/genes8120350. 29182580 PMC5748668

[47] Gillam EM, Notley LM, Cai H, De Voss JJ, Guengerich FP 2000 Oxidation of indole by cytochrome P450 enzymes. Biochemistry 39:13817–13824. doi:10.1021/bi001229u. 11076521

[48] Madsen EL, Bollag JM 1988 Pathway of indole metabolism by a denitrifying microbial community. Arch Microbiol 151:71–76. doi:10.1007/BF00444672.

[49] Claus SP, Guillou H, Ellero-Simatos S 2016 The gut microbiota: a major player in the toxicity of environmental pollutants? NPJ Biofilms Microbiomes 2:16003. doi:10.1038/npjbiofilms.2016.3. 28721242 PMC5515271

[50] Koch HM, Preuss R, Angerer J 2006 Di(2-ethylhexyl)phthalate (DEHP): human metabolism and internal exposure– an update and latest results. Int J Androl 29:155–165. doi:10.1111/j.1365-2605.2005.00607.x. 16466535

[51] De Angelis M, Piccolo M, Vannini L, Siragusa S, De Giacomo A, Serrazzanetti DI, Cristofori F, Guerzoni ME, Gobbetti M, Francavilla R 2013 Fecal microbiota and metabolome of children with autism and pervasive developmental disorder not otherwise specified. PLoS One 8:e76993. doi:10.1371/journal.pone.0076993. 24130822 PMC3793965

[52] Gabriele S, Sacco R, Cerullo S, Neri C, Urbani A, Tripi G, Malvy J, Barthelemy C, Bonnet-Brihault F, Persico AM 2014 Urinary *p*-cresol is elevated in young French children with autism spectrum disorder: a replication study. Biomarkers 19:463–470. doi:10.3109/1354750X.2014.936911. 25010144

[53] Bourassa MW, Alim I, Bultman SJ, Ratan RR 2016 Butyrate, neuroepigenetics and the gut microbiome: can a high fiber diet improve brain health? Neurosci Lett 625:56–63. doi:10.1016/j.neulet.2016.02.009. 26868600 PMC4903954

[54] Selmer T, Andrei PI 2001 *p*-Hydroxyphenylacetate decarboxylase from *Clostridium difficile*. A novel glycyl radical enzyme catalysing the formation of *p*-cresol. Eur J Biochem 268:1363–1372. doi:10.1046/j.1432-1327.2001.02001.x. 11231288

[55] Saito Y, Sato T, Nomoto K, Tsuji H 2018 Identification of phenol- and *p*-cresol-producing intestinal bacteria by using media supplemented with tyrosine and its metabolites. FEMS Microbiol Ecol 94:fiy125. doi:10.1093/femsec/fiy125. PMC6424909 29982420

[56] Fujisaka S, Avila-Pacheco J, Soto M, Kostic A, Dreyfuss JM, Pan H, Ussar S, Altindis E, Li N, Bry L, Clish CB, Kahn CR 2018 Diet, genetics, and the gut microbiome drive dynamic changes in plasma metabolites. Cell Rep 22:3072–3086. doi:10.1016/j.celrep.2018.02.060. 29539432 PMC5880543

[57] Franzosa EA, Sirota-Madi A, Avila-Pacheco J, Fornelos N, Haiser HJ, Reinker S, Vatanen T, Hall AB, Mallick H, McIver LJ, Sauk JS, Wilson RG, Stevens BW, Scott JM, Pierce K, Deik AA, Bullock K, Imhann F, Porter JA, Zhernakova A, Fu J, Weersma RK, Wijmenga C, Clish CB, Vlamakis H, Huttenhower C, Xavier RJ 2019 Gut microbiome structure and metabolic activity in inflammatory bowel disease. Nat Microbiol 4:293–305. doi:10.1038/s41564-018-0306-4. 30531976 PMC6342642

[58] Alden N, Krishnan S, Porokhin V, Raju R, McElearney K, Gilbert A, Lee K 2017 Biologically consistent annotation of metabolomics data. Anal Chem 89:13097–13104. doi:10.1021/acs.analchem.7b02162. 29156137

[59] Smith CA, O'Maille G, Want EJ, Qin C, Trauger SA, Brandon TR, Custodio DE, Abagyan R, Siuzdak G 2005 METLIN: a metabolite mass spectral database. Ther Drug Monit 27:747–751. doi:10.1097/01.ftd.0000179845.53213.39. 16404815

[60] Wishart DS, Tzur D, Knox C, Eisner R, Guo AC, Young N, Cheng D, Jewell K, Arndt D, Sawhney S, Fung C, Nikolai L, Lewis M, Coutouly MA, Forsythe I, Tang P, Shrivastava S, Jeroncic K, Stothard P, Amegbey G, Block D, Hau DD, Wagner J, Miniaci J, Clements M, Gebremedhin M, Guo N, Zhang Y, Duggan GE, Macinnis GD, Weljie AM, Dowlatabadi R, Bamforth F, Clive D, Greiner R, Li L, Marrie T, Sykes BD, Vogel HJ, Querengesser L 2007 HMDB: the human metabolome database. Nucleic Acids Res 35:D521–D526. doi:10.1093/nar/gkl923. 17202168 PMC1899095

[61] Johnson S 2018 NIST standard reference database 1A v17. National Institute of Standards and Technology, Gaithersburg, MD.

[62] Wolf S, Schmidt S, Muller-Hannemann M, Neumann S 2010 *In silico* fragmentation for computer assisted identification of metabolite mass spectra. BMC Bioinformatics 11:148. doi:10.1186/1471-2105-11-148. 20307295 PMC2853470

[63] Allen F, Pon A, Wilson M, Greiner R, Wishart D 2014 CFM-ID: a web server for annotation, spectrum prediction and metabolite identification from tandem mass spectra. Nucleic Acids Res 42:W94–W99. doi:10.1093/nar/gku436. 24895432 PMC4086103

[64] Manteiga S, Lee K 2017 Monoethylhexyl phthalate elicits an inflammatory response in adipocytes characterized by alterations in lipid and cytokine pathways. Environ Health Perspect 125:615–622. doi:10.1289/EHP464. 27384973 PMC5381996

[65] Kozich JJ, Westcott SL, Baxter NT, Highlander SK, Schloss PD 2013 Development of a dual-index sequencing strategy and curation pipeline for analyzing amplicon sequence data on the MiSeq Illumina sequencing platform. Appl Environ Microbiol 79:5112–5120. doi:10.1128/AEM.01043-13. 23793624 PMC3753973

[66] Edgar RC, Haas BJ, Clemente JC, Quince C, Knight R 2011 UCHIME improves sensitivity and speed of chimera detection. Bioinformatics 27:2194–2200. doi:10.1093/bioinformatics/btr381. 21700674 PMC3150044

[67] Caporaso JG, Kuczynski J, Stombaugh J, Bittinger K, Bushman FD, Costello EK, Fierer N, Pena AG, Goodrich JK, Gordon JI, Huttley GA, Kelley ST, Knights D, Koenig JE, Ley RE, Lozupone CA, McDonald D, Muegge BD, Pirrung M, Reeder J, Sevinsky JR, Turnbaugh PJ, Walters WA, Widmann J, Yatsunenko T, Zaneveld J, Knight R 2010 QIIME allows analysis of high-throughput community sequencing data. Nat Methods 7:335–336. doi:10.1038/nmeth.f.303. 20383131 PMC3156573

[68] Kanehisa M, Goto S 2000 KEGG: Kyoto encyclopedia of genes and genomes. Nucleic Acids Res 28:27–30. doi:10.1093/nar/28.1.27. 10592173 PMC102409

[69] Lloyd-Price J, Mahurkar A, Rahnavard G, Crabtree J, Orvis J, Hall AB, Brady A, Creasy HH, McCracken C, Giglio MG, McDonald D, Franzosa EA, Knight R, White O, Huttenhower C 2017 Strains, functions and dynamics in the expanded Human Microbiome Project. Nature 550:61–66. doi:10.1038/nature23889. 28953883 PMC5831082

[70] Kanehisa M, Sato Y, Morishima K 2016 BlastKOALA and GhostKOALA: KEGG tools for functional characterization of genome and metagenome sequences. J Mol Biol 428:726–731. doi:10.1016/j.jmb.2015.11.006. 26585406

[71] Segata N, Izard J, Waldron L, Gevers D, Miropolsky L, Garrett WS, Huttenhower C 2011 Metagenomic biomarker discovery and explanation. Genome Biol 12:R60. doi:10.1186/gb-2011-12-6-r60. 21702898 PMC3218848

[72] Benjamini Y, Hochberg Y 1995 Controlling the false discovery rate: a practical and powerful approach to multiple testing. J R Stat Soc Series B Stat Methodol 57:289–300. doi:10.1111/j.2517-6161.1995.tb02031.x.

