# Supplementary material for: Environmental Chemical Diethylhexyl Phthalate Alters Intestinal Microbiota Community Structure and Metabolite Profile in Mice
Source: mSystems. 2019 Dec 10;4(6):e00724-19. doi: 10.1128/mSystems.00724-19 (PMC6906742; doi:10.1128/mSystems.00724-19)
Supplement: TABLE S1 [file mSystems.00724-19-st001.docx]

Table S1. Putatively identified compounds in cecal culture

| **Mode** | **m/z** | **RT** | **KEGG ID** | **Name** |
| --- | --- | --- | --- | --- |
| + | 75.0434 | 4.14 | C17530 | Methyl acetate |
| + | 86.0595 | 5.93 | C02659 | Acetone cyanohydrin |
| + | 89.0588 | 7.27 | C00810 | (R)-Acetoin |
| + | 100.0761 | 8.26 | C18796 | (2R)-2-Hydroxy-2-methylbutanenitrile |
| + | 104.0697 | 22.63 | C00334 | 4-Aminobutanoate |
| + | 107.0488 | 11.46 | C00261 | Benzaldehyde |
| + | 109.0641 | 16.96 | C01468 | 4-Cresol |
| + | 115.0495 | 6.29 | C00429 | 5,6-Dihydrouracil |
| + | 115.0744 | 18.97 | C01880 | epsilon-Caprolactone |
| + | 116.0698 | 4.03 | C00148 | L-Proline |
| + | 117.0562 | 6.78 | C03273 | 5-Oxopentanoate |
| + | 118.0662 | 23.8 | C00463 | Indole |
| + | 118.0856 | 4.2 | C00431 | 5-Aminopentanoate |
| + | 122.0962 | 4.93 | C05332 | Phenethylamine |
| + | 124.0381 | 4.08 | C00253 | Nicotinate |
| + | 128.07 | 4.09 | C00450 | (S)-2,3,4,5-Tetrahydropyridine-2-carboxylate |
| + | 130.0644 | 20.9 | C06323 | Isoquinoline |
| + | 130.0849 | 4.07 | C05936 | N4-Acetylaminobutanal |
| + | 131.1171 | 4.03 | C02714 | N-Acetylputrescine |
| + | 132.0647 | 6.71 | C01165 | L-Glutamate 5-semialdehyde |
| + | 132.1 | 4.08 | C00407 | L-Isoleucine |
| + | 134.0598 | 18.58 | C00561 | Mandelonitrile |
| + | 136.0607 | 4.42 | C00147 | Adenine |
| + | 137.0452 | 4.34 | C00262 | Hypoxanthine |
| + | 137.0598 | 17.09 | C07086 | Phenylacetic acid |
| + | 138.0529 | 4.27 | C00108 | Anthranilate |
| + | 141.0646 | 3.99 | C20522 | Dihydrourocanate |
| + | 142.0279 | 6.22 | C05678 | (2-Amino-1-hydroxyethyl)phosphonate |
| + | 145.0482 | 4.36 | C00922 | 2,3-Dimethylmaleate |
| + | 146.0601 | 6.8 | C06324 | Isocarbostyril |
| + | 146.1638 | 4.02 | C00315 | Spermidine |
| + | 147.0418 | 4.12 | C20231 | (Z)-3-Ureidoacrylate peracid |
| + | 148.0388 | 26 | C05579 | Indole-5,6-quinone |
| + | 149.0583 | 17.47 | C00423 | trans-Cinnamate |
| + | 150.0575 | 4.03 | C00073 | L-Methionine |
| + | 151.0394 | 4.14 | C02137 | alpha-Oxo-benzeneacetic acid |
| + | 160.1813 | 4.02 | C06366 | sym-Homospermidine |
| + | 161.1056 | 6.22 | C00398 | Tryptamine |
| + | 162.0543 | 19.24 | C05639 | 4,6-Dihydroxyquinoline |
| + | 165.0533 | 4.14 | C00166 | Phenylpyruvate |
| + | 166.0852 | 4.91 | C00079 | L-Phenylalanine |
| + | 169.085 | 17.27 | C06459 | N-Trimethyl-2-aminoethylphosphonate |
| + | 175.0872 | 4.22 | C02693 | (Indol-3-yl)acetamide |
| + | 175.1184 | 3.99 | C00062 | L-Arginine |
| + | 176.0702 | 20.82 | C00954 | Indole-3-acetate |
| + | 177.0643 | 5.21 | C00499 | Allantoate |
| + | 177.1016 | 4.35 | C00780 | Serotonin |
| + | 181.0973 | 18.25 | C05638 | 5-Hydroxykynurenamine |
| + | 182.0815 | 4.11 | C00082 | L-Tyrosine |
| + | 183.0845 | 4.27 | C00794 | D-Sorbitol |
| + | 187.0231 | 32.25 | C02514 | 3-Fumarylpyruvate |
| + | 188.1267 | 17.4 | C01092 | 8-Amino-7-oxononanoate |
| + | 190.0505 | 4.12 | C14800 | 1-Nitronaphthalene-5,6-oxide |
| + | 196.0631 | 23.42 | C00822 | Dopaquinone |
| + | 201.0422 | 23.36 | C04642 | 2-Hydroxy-5-carboxymethylmuconate semialdehyde |
| + | 204.0657 | 18.77 | C00331 | Indolepyruvate |
| + | 205.0979 | 6.84 | C00078 | L-Tryptophan |
| + | 213.159 | 22.87 | C16836 | 1,4'-Bipiperidine-1'-carboxylic acid |
| + | 215.1398 | 4.29 | C01909 | Dethiobiotin |
| + | 217.061 | 5.16 | C02954 | 6-Aminopenicillanate |
| + | 217.1185 | 4.08 | C15700 | gamma-Glutamyl-gamma-aminobutyraldehyde |
| + | 218.1161 | 24.51 | C15532 | N-Acetyl-L-citrulline |
| + | 221.0922 | 15.8 | C00643 | 5-Hydroxy-L-tryptophan |
| + | 233.1253 | 20.07 | C01598 | Melatonin |
| + | 243.1018 | 27.44 | C21418 | (2R,3S,4S)-Leucoanthocyanidin |
| + | 245.0946 | 6.68 | C00120 | Biotin |
| + | 249.1206 | 15.3 | C05643 | 6-Hydroxymelatonin |
| + | 252.109 | 4.69 | C00559 | Deoxyadenosine |
| + | 257.0913 | 4.03 | C05841 | Nicotinate D-ribonucleoside |
| + | 268.1052 | 4.48 | C00212 | Adenosine |
| + | 272.1257 | 27.42 | C11785 | Normorphine |
| + | 277.1175 | 6.82 | C04732 | 5-Amino-6-(1-D-ribitylamino)uracil |
| + | 277.1426 | 29.66 | C00449 | N6-(L-1,3-Dicarboxypropyl)-L-lysine |
| + | 279.1347 | 5.47 | C00831 | Pantetheine |
| + | 284.0995 | 5.11 | C00387 | Guanosine |
| + | 291.0438 | 6.6 | C05382 | Sedoheptulose 7-phosphate |
| + | 295.0831 | 17.32 | C17017 | Cyclic dehypoxanthine futalosine |
| + | 296.0668 | 3.99 | C03373 | Aminoimidazole ribotide |
| + | 298.0981 | 8.65 | C00170 | 5'-Methylthioadenosine |
| + | 299.0813 | 17.63 | C19787 | 5'-S-Methyl-5'-thioinosine |
| + | 300.1555 | 4.14 | C06174 | Codeine |
| + | 316.1506 | 23 | C05202 | 3'-Hydroxy-N-methyl-(S)-coclaurine |
| + | 324.1658 | 4.06 | C20910 | L-4-Hydroxyphenylglycyl-L-arginine |
| + | 336.139 | 16.09 | C04540 | N4-(Acetyl-beta-D-glucosaminyl)asparagine |
| + | 339.0678 | 5.25 | C04677 | 1-(5'-Phosphoribosyl)-5-amino-4-imidazolecarboxamide |
| + | 347.1963 | 4.27 | C21160 | L-Alanyl-gamma-D-glutamyl-L-lysine |
| + | 352.1239 | 5.01 | C04015 | N-Acetyl-4-O-acetylneuraminate |
| + | 355.0633 | 24.2 | C01268 | 5-Amino-6-(5'-phosphoribosylamino)uracil |
| + | 365.1573 | 18.68 | C02999 | N-Acetylmuramoyl-Ala |
| + | 377.1476 | 20.75 | C00255 | Riboflavin |
| + | 393.3005 | 36.73 | C04483 | Deoxycholic acid |
| + | 407.2789 | 32.49 | C04643 | 7-Oxodeoxycholate |
| + | 450.3272 | 35.46 | C05464 | Glycodeoxycholate |
| + | 466.3199 | 33.39 | C01921 | Glycocholate |
| + | 516.2924 | 29.06 | C05122 | Taurocholate |
| + | 587.2899 | 4.48 | C16641 | Irinotecan |
| - | 88.0415 | 9.89 | C00041 | L-Alanine |
| - | 89.0244 | 8.08 | C00256 | (R)-Lactate |
| - | 94.9817 | 20.22 | C11145 | Methanesulfonic acid |
| - | 102.0565 | 15.5 | C00334 | 4-Aminobutanoate |
| - | 103.0398 | 11.77 | C05984 | 2-Hydroxybutanoic acid |
| - | 104.0344 | 11.12 | C00065 | L-Serine |
| - | 107.0491 | 15.6 | C01468 | 4-Cresol |
| - | 111.0206 | 5.79 | C00106 | Uracil |
| - | 113.0244 | 19.46 | C00596 | 2-Hydroxy-2,4-pentadienoate |
| - | 114.0571 | 9.5 | C00148 | L-Proline |
| - | 119.035 | 12.75 | C15587 | Purine |
| - | 121.0291 | 8.73 | C00633 | 4-Hydroxybenzaldehyde |
| - | 121.0408 | 34.2 | C00153 | Nicotinamide |
| - | 122.0244 | 13.16 | C00253 | Nicotinate |
| - | 124.0076 | 10.16 | C00245 | Taurine |
| - | 128.035 | 15.48 | C01879 | Pidolic acid |
| - | 129.0554 | 9.34 | C00671 | (S)-3-Methyl-2-oxopentanoic acid |
| - | 130.0892 | 8.34 | C00407 | L-Isoleucine |
| - | 131.0349 | 22.79 | C03589 | 4-Hydroxy-2-oxopentanoate |
| - | 131.0711 | 9.66 | C06103 | 6-Hydroxyhexanoic acid |
| - | 135.0318 | 9.57 | C00262 | Hypoxanthine |
| - | 135.0454 | 10.82 | C07086 | Phenylacetic acid |
| - | 137.0346 | 15.69 | C00785 | Urocanate |
| - | 139.0522 | 15.18 | C20522 | Dihydrourocanate |
| - | 143.0332 | 8.08 | C00922 | 2,3-Dimethylmaleate |
| - | 145.098 | 11.85 | C00047 | L-Lysine |
| - | 146.0453 | 15.5 | C00025 | L-Glutamate |
| - | 147.0658 | 9.93 | C06007 | (R)-2,3-Dihydroxy-3-methylpentanoate |
| - | 148.0459 | 9.33 | C00073 | L-Methionine |
| - | 149.0466 | 13.65 | C00121 | D-Ribose |
| - | 149.0605 | 9.58 | C05629 | Phenylpropanoate |
| - | 150.0554 | 14.64 | C07301 | (R)-Mandelamide |
| - | 151.028 | 14.43 | C00385 | Xanthine |
| - | 151.0398 | 15.58 | C00642 | 4-Hydroxyphenylacetate |
| - | 154.0491 | 9.69 | C19830 | (5S,6S)-6-Amino-5-hydroxycyclohexa-1,3-diene-1-carboxylate |
| - | 154.0618 | 11.12 | C00135 | L-Histidine |
| - | 155.0339 | 12.61 | C06210 | 2-Hydroxy-6-keto-2,4-heptadienoate |
| - | 160.0397 | 7.23 | C05639 | 4,6-Dihydroxyquinoline |
| - | 160.0626 | 19.41 | C01077 | O-Acetyl-L-homoserine |
| - | 161.0447 | 9.59 | C03979 | 2-Dehydro-3-deoxy-L-rhamnonate |
| - | 161.0951 | 11.46 | C01028 | N6-Hydroxy-L-lysine |
| - | 163.038 | 10.12 | C00166 | Phenylpyruvate |
| - | 164.0356 | 11.12 | C05653 | Formylanthranilate |
| - | 164.0735 | 9.12 | C00079 | L-Phenylalanine |
| - | 165.0197 | 22.69 | C03590 | 4-Hydroxyphenylglyoxylate |
| - | 165.0405 | 23.94 | C16358 | 1-Methylxanthine |
| - | 165.0569 | 14.19 | C11457 | 3-(3-Hydroxyphenyl)propanoic acid |
| - | 167.0331 | 9.35 | C06672 | Vanillate |
| - | 171.0075 | 12.64 | C00093 | sn-Glycerol 3-phosphate |
| - | 171.0275 | 12.88 | C02637 | 3-Dehydroshikimate |
| - | 173.0585 | 13.2 | C00439 | N-Formimino-L-glutamate |
| - | 173.1027 | 10.82 | C00062 | L-Arginine |
| - | 174.0392 | 13.31 | C01045 | N-Formyl-L-glutamate |
| - | 178.0703 | 10.9 | C00329 | D-Glucosamine |
| - | 179.056 | 8.08 | C00031 | D-Glucose |
| - | 180.0659 | 10.12 | C00082 | L-Tyrosine |
| - | 183.065 | 10.15 | C11588 | cis-3-(Carboxy-ethyl)-3,5-cyclo-hexadiene-1,2-diol |
| - | 184 | 14.8 | C01005 | O-Phospho-L-serine |
| - | 186.1125 | 9.48 | C01092 | 8-Amino-7-oxononanoate |
| - | 189.0531 | 9.59 | C05829 | N-Carbamyl-L-glutamate |
| - | 191.0169 | 20.69 | C00679 | 5-Dehydro-4-deoxy-D-glucarate |
| - | 195.0504 | 14.54 | C00257 | D-Gluconic acid |
| - | 195.9906 | 22.3 | C05335 | L-Selenomethionine |
| - | 196.0604 | 9.57 | C00355 | 3,4-Dihydroxy-L-phenylalanine |
| - | 196.0994 | 9.04 | C05588 | L-Metanephrine |
| - | 198.0763 | 10.82 | C20941 | L-Anticapsin |
| - | 203.0847 | 9.31 | C00078 | L-Tryptophan |
| - | 203.1011 | 11.34 | C04210 | N5-(L-1-Carboxyethyl)-L-ornithine |
| - | 211.0243 | 9.95 | C12624 | 2-Hydroxy-6-ketononatrienedioate |
| - | 213.1241 | 9.71 | C01909 | Dethiobiotin |
| - | 214.048 | 10.89 | C01233 | sn-Glycero-3-phosphoethanolamine |
| - | 216.0966 | 12.74 | C15532 | N-Acetyl-L-citrulline |
| - | 216.133 | 11.07 | C15699 | gamma-L-Glutamylputrescine |
| - | 226.0089 | 13.04 | C03287 | L-Glutamyl 5-phosphate |
| - | 226.0745 | 8.54 | C00826 | L-Arogenate |
| - | 226.0802 | 5.13 | C00881 | Deoxycytidine |
| - | 231.0957 | 21.37 | C15767 | 4-(L-gamma-Glutamylamino)butanoate |
| - | 239.074 | 8.88 | C21417 | (2R,3R)-Dihydroflavonol |
| - | 243.0157 | 19.63 | C20829 | Sulfoquinovose |
| - | 243.0588 | 6.32 | C00299 | Uridine |
| - | 259.025 | 21.82 | C05345 | beta-D-Fructose 6-phosphate |
| - | 259.0725 | 5.05 | C20386 | Biotin sulfoxide |
| - | 264.1064 | 7.06 | C00378 | Thiamine |
| - | 270.1148 | 10.65 | C11785 | Normorphine |
| - | 272.1654 | 9.58 | C20279 | (2R,3R)-3-Methylglutamyl-5-semialdehyde-N6-lysine |
| - | 274.1017 | 19.38 | C18048 | N-Succinyl-L-citrulline |
| - | 275.0969 | 10.71 | C04732 | 5-Amino-6-(1-D-ribitylamino)uracil |
| - | 275.122 | 15.75 | C00449 | N6-(L-1,3-Dicarboxypropyl)-L-lysine |
| - | 279.2362 | 7.98 | C01595 | Linoleate |
| - | 281.2527 | 8.05 | C00712 | (9Z)-Octadecenoic acid |
| - | 282.0814 | 9.66 | C00387 | Guanosine |
| - | 283.071 | 20.84 | C01762 | Xanthosine |
| - | 288.1222 | 21.45 | C21016 | Ophthalmate |
| - | 294.0973 | 9.99 | C00844 | Prunasin |
| - | 295.0802 | 9.75 | C17010 | Dehypoxanthine futalosine |
| - | 297.0678 | 10.49 | C19787 | 5'-S-Methyl-5'-thioinosine |
| - | 299.2634 | 8.47 | C03195 | (R)-10-Hydroxystearate |
| - | 309.1448 | 9.62 | C16608 | Demethylcitalopram |
| - | 321.1084 | 9.64 | C04079 | N-((R)-Pantothenoyl)-L-cysteine |
| - | 323.1539 | 9.69 | C07572 | Citalopram |
| - | 333.0472 | 10.6 | C00455 | Nicotinamide D-ribonucleotide |
| - | 335.0455 | 9.96 | C01185 | Nicotinate D-ribonucleotide |
| - | 337.0539 | 14.01 | C04677 | 1-(5'-Phosphoribosyl)-5-amino-4-imidazolecarboxamide |
| - | 338.0365 | 9.19 | C04751 | 1-(5-Phospho-D-ribosyl)-5-amino-4-imidazolecarboxylate |
| - | 341.1081 | 9.69 | C00089 | Sucrose |
| - | 343.1273 | 9.7 | C05399 | Melibiitol |
| - | 344.0742 | 10.16 | C01081 | Thiamin monophosphate |
| - | 352.0676 | 20.62 | C01304 | 2,5-Diamino-6-(5-phospho-D-ribosylamino)pyrimidin-4(3H)-one |
| - | 357.0936 | 19.39 | C01134 | Pantetheine 4'-phosphate |
| - | 365.0462 | 19.59 | C04734 | 1-(5'-Phosphoribosyl)-5-formamido-4-imidazolecarboxamide |
| - | 369.0039 | 14.74 | C00447 | Sedoheptulose 1,7-bisphosphate |
| - | 375.1331 | 9.66 | C00255 | Riboflavin |
| - | 383.1151 | 10.14 | C00021 | S-Adenosyl-L-homocysteine |
| - | 391.2895 | 10.06 | C04483 | Deoxycholic acid |
| - | 407.2809 | 14.73 | C00695 | Cholic acid |
| - | 440.1379 | 19.65 | C00504 | Folate |
| - | 442.1544 | 19.39 | C00415 | Dihydrofolate |
| - | 444.1646 | 9.64 | C00101 | Tetrahydrofolate |
| - | 456.165 | 9.68 | C00143 | 5,10-Methylenetetrahydrofolate |
| - | 472.1146 | 9.78 | C05674 | CMP-N-trimethyl-2-aminoethylphosphonate |
| - | 498.2958 | 13 | C05463 | Taurodeoxycholate |
| - | 503.1582 | 10.44 | C00492 | Raffinose |
| - | 530.0624 | 10.17 | C04297 | CDP-4-dehydro-3,6-dideoxy-D-glucose |
| - | 564.0644 | 9.57 | C00501 | CDP-glucose |
| - | 581.239 | 19.38 | C00500 | Biliverdin |
| - | 583.2611 | 19.38 | C00486 | Bilirubin |
| - | 590.0837 | 10.14 | C20768 | UDP-2-acetamido-2,6-dideoxy-beta-L-talose |
| - | 606.0735 | 9.61 | C00043 | UDP-N-acetyl-alpha-D-glucosamine |
| - | 631.2507 | 9.55 | C18154 | 3-Hydroxyethylchlorophyllide a |
| - | 637.1041 | 10.52 | C12632 | Luteolin 7-O-[beta-D-glucuronosyl-(1->2)-beta-D-glucuronide] |
| - | 665.2187 | 9.62 | C01613 | Stachyose |
| - | 813.1488 | 9.74 | C04900 | Luteolin 7-O-[beta-D-glucuronosyl-(1->2)-beta-D-glucuronide]-4'-O-beta-D-glucuronide |
| - | 832.2563 | 9.31 | C04738 | UDP-3-O-(3-hydroxytetradecanoyl)-N-acetylglucosamine |
| - | 852.1527 | 10.84 | C01144 | (S)-3-Hydroxybutanoyl-CoA |
| - | 888.0675 | 9.94 | C19685 | Sulfoacetyl-CoA |
