# Supplementary material for: Environmental Chemical Diethylhexyl Phthalate Alters Intestinal Microbiota Community Structure and Metabolite Profile in Mice
Source: mSystems. 2019 Dec 10;4(6):e00724-19. doi: 10.1128/mSystems.00724-19 (PMC6906742; doi:10.1128/mSystems.00724-19)
Supplement: TABLE S2 [file mSystems.00724-19-st002.docx]

Table S2. Confidently identified (level 2) and confirmed metabolites (level 1).

| **Ionization mode** | **m/z** | **RT** | **KEGG ID** | **Name** | **Level** |
| --- | --- | --- | --- | --- | --- |
| + | 60.0822 | 4.06 | C00565 | trimethylamine | 2 |
| + | 75.0434 | 4.14 | C00163 | propionic acid | 1 |
| + | 89.0588 | 7.27 | C00246 | butyric acid | 1 |
| + | 104.1056 | 3.99 | C00114 | choline | 2 |
| + | 107.0487 | 16.76 | C00261 | benzaldehyde | 1 |
| + | 109.0641 | 16.96 | C01468 | p-cresol | 1 |
| + | 118.0662 | 23.8 | C00463 | indole | 1 |
| + | 118.0856 | 4.2 | C00719 | betaine | 2 |
| + | 121.0628 | 17.59 | C00601 | phenylacetaldehyde | 2 |
| + | 122.0962 | 4.93 | C05332 | phenylethylamine | 1 |
| + | 123.0559 | 4.42 | C00153 | nicotinamide | 2 |
| + | 124.0381 | 4.08 | C00253 | naicin | 2 |
| + | 131.1171 | 4.03 | C02714 | N-acetylputrescine | 2 |
| + | 132.1016 | 4.08 | C00407 | isoleucine | 1 |
| + | 134.0598 | 18.58 | C05658 | indoxyl | 2 |
| + | 137.0452 | 4.34 | C00262 | hypoxanthine | 1 |
| + | 137.0598 | 17.09 | C07086 | phenylacetic acid | 1 |
| + | 141.0646 | 3.99 | C05828 | methylimidazole acetic acid | 2 |
| + | 146.1159 | 4.14 | C01181 | gamma-butyrobetaine | 2 |
| + | 161.1056 | 6.22 | C00398 | tryptamine | 1 |
| + | 162.1131 | 3.98 | C00487 | carnitine | 2 |
| + | 166.0852 | 4.91 | C00079 | phenylalanine | 1 |
| + | 176.0702 | 20.82 | C00954 | indole-3-acetate | 1 |
| + | 177.1016 | 4.35 | C00780 | serotonin | 1 |
| + | 188.1751 | 4.04 | C00612 | N1-acetylspermidine | 2 |
| + | 190.0849 | 24.35 | NA | 3-indolepropionic acid | 2 |
| + | 204.0657 | 18.77 | C00331 | indolepyruvate | 2 |
| + | 205.0979 | 6.84 | C00078 | tryptophan | 1 |
| + | 243.0881 | 24.98 | C01727 | lumichrome | 2 |
| + | 243.1018 | 27.44 | C14131 | equol | 2 |
| + | 252.1205 | 17.37 | C01657 | N-acetyl-L-tyrosine ethyl ester | 2 |
| + | 377.1476 | 20.75 | C00255 | riboflavin (Vitamin B2) | 2 |
| - | 89.0267 | 13.19 | C00186 | lactate | 1 |
| - | 104.0344 | 11.12 | C00065 | serine | 1 |
| - | 111.0206 | 5.79 | C00106 | uracil | 1 |
| - | 114.0571 | 9.5 | C00148 | proline | 1 |
| - | 124.0076 | 10.16 | C00245 | taurine | 1 |
| - | 131.0711 | 9.66 | NA | leucic acid | 2 |
| - | 145.098 | 11.85 | C00047 | lysine | 1 |
| - | 146.0453 | 15.5 | C00025 | glutamate | 1 |
| - | 148.0459 | 9.33 | C00073 | methionine | 1 |
| - | 149.0605 | 9.58 | C05629 | 3-phenylpropanoic acid | 2 |
| - | 151.028 | 14.43 | C00385 | xanthine | 1 |
| - | 151.0398 | 15.58 | C00642 | p-hydroxyphenylacetic acid | 1 |
| - | 154.0618 | 11.12 | C00135 | histidine | 1 |
| - | 165.0569 | 14.19 | C11457 | 3-(3-hydroxyphenyl)propionic acid | 1 |
| - | 173.1027 | 10.82 | C00062 | arginine | 1 |
| - | 180.0659 | 10.12 | C00082 | tyrosine | 1 |
| - | 195.0504 | 14.54 | C00257 | gluconic acid | 2 |
| - | 243.0588 | 6.32 | C00299 | uridine | 1 |
| - | 255.2354 | 8.2 | C00249 | palmitic acid | 1 |
| - | 277.1436 | 8.63 | C03343 | monoethylhexyl phthalate | 1 |
| - | 281.2527 | 8.05 | C00712 | oleic acid | 1 |
| - | 282.0814 | 9.66 | C00387 | guanosine | 2 |
| - | 283.2634 | 7.77 | C01530 | stearic acid | 2 |
| - | 299.2634 | 8.47 | C03195 | 2-hydroxystearic acid | 2 |
| - | 391.2895 | 10.06 | C04483 | deoxycholic acid | 2 |

Confident identification (level 2) refers to metabolites matching a standard by at least two of the following measures: accurate mass, MS/MS (external reference standards), and retention time (RT). Confirmed identification (level 1) refers to metabolites matching a standard by at least two of the following measures: accurate mass, MS/MS (internal reference standards), and retention time (RT).
