# Supplementary material for: Environmental Chemical Diethylhexyl Phthalate Alters Intestinal Microbiota Community Structure and Metabolite Profile in Mice
Source: mSystems. 2019 Dec 10;4(6):e00724-19. doi: 10.1128/mSystems.00724-19 (PMC6906742; doi:10.1128/mSystems.00724-19)
Supplement: TEXT S1 [file mSystems.00724-19-s0001.docx]

**Supplementary Methods**

***Lachnoclostridium bolteae* ATCC BAA-613 monoculture**

*L. bolteae* ATCC BAA-613 was cultured in 5 ml of GMM or tryptic soy broth (TSB) at 37°C for 48 hours in an anaerobic chamber (Coy). The GMM cultures were supplemented with 100 μM DEHP or vehicle. The TSB cultures were supplemented with 200 μM tyrosine (Sigma), 100 μM DEHP, both or vehicle. Negative controls (GMM or TSB without *L. bolteae*) were incubated under identical conditions as the cultures to confirm that any change in metabolite concentration was due to bacterial metabolic activity. Samples were taken at 48 hours and stored at -80°C until analysis. Metabolites were extracted as per the protocol for untargeted analysis (same as the GMM *in vitro* study). The extracts were lyophilized and resuspended in 50% MeOH/water (HPLC grade).

**LC-MS**

The IDA experiments comprised a TOF MS (survey) scan and (triggered) high-resolution MS/MS (product ion) scans monitoring up to 8 candidate ions per cycle. The dependent scans were triggered whenever the survey scan detected a precursor ion with an exact mass in the range of m/z 40-1500. The mass tolerance was set to 50 mDa. Precursor ions having the same mass as a previously fragmented precursor ion were excluded from fragmentation for a 5 sec window to increase the probability of fragmenting different ions. Dynamic background subtraction was applied to improve detection of precursor ions. The positive mode experiments were paired with a gradient based liquid chromatography (LC) method that uses a C18 reverse phase (RP) column (Synergi Fusion-RP 4µ 80Å 150 x 2.0 mm, Phenomenex, Torrance, CA). Solvent A was water + 0.1% formic acid and solvent B was methanol + 0.1% formic acid. The mobile phase gradient was as follows. The flow rate was initially at 100 µL/min. Solvent A was held at 97% from 0 to 8 min. The flow rate was changed to 300 µL/min. Solvent A was linearly decreased to 5% over 30 min, held at 5% for 7 min, and linearly increased to 97% over 2 min. The flow rate was changed to 100 µL/min and held at 97% for 8 min. The negative mode experiments were paired with a gradient based method that uses a hydrophilic interaction chromatography (HILIC) column (Luna NH_2_ 5µ 100Å 250 x 2.0 mm, Phenomenex). Solvent A was a 5% acetonitrile solution in water (v/v) + 20 mM ammonium hydroxide and 20 mM ammonium acetate (pH 9.45). Solvent B was acetonitrile. The flow rate was held constant at 300 µL/min. The mobile phase gradient was as follows. Solvent A was initially set at 15%, linearly increased to 100% over 15 min, held at 100% for 13 min, linearly decreased to 15% over 2 min, and held at 15% for 20 min. Both LC methods used an injection volume of 15  μl. Samples entering the mass analyzer were ionized using a DuoSpray ion source (TurboIonSpray probe, AB Sciex).
